# Diverse ancestral myosin motors generate and segregate distinct types of nanocluster-rich domains at the plasma membrane

**DOI:** 10.1126/sciadv.aeb8889

**Published:** 2026-09-11

**Authors:** Parijat Sil, Thomas S. van Zanten, Sowmya Jahnavi, Ajay Bansal, Hafez Razmazma, Suvrajit Saha, Bhagyashri Mahajan, Mukesh Kumar, Phillip J. Stansfeld, Parvinder Pal Singh, Madan Rao, Satyajit Mayor

**Affiliations:** ^1^ National Centre for Biological Sciences, Bangalore, India.; ^2^ Fundación Agencia Aragonesa para la Investigación y el Desarrollo (ARAID), Instituto de Nanociencia y Materiales de Aragón (INMA), CSIC-Universidad de Zaragoza and CIBER-BBN, Zaragoza, Spain.; ^3^ Warwick Medical School, University of Warwick, Coventry, UK.; ^4^ Simons Centre for the Study of Living Machines, National Centre for Biological Sciences, Bangalore, India.; ^5^ School of Life Sciences and Department of Chemistry, University of Warwick, Coventry, UK.; ^6^ Trans-disciplinary University, Bangalore, India.; ^7^ CSIR–Indian Institute of Integrative Medicine, Jammu, India.

## Abstract

Molecular organization of the plasma membrane at nano- and micrometer scales is critical for its function in all living cells. This emerges not only from the self-assembly of lipids and proteins but also from active forces originating in the underlying cytoskeletal cortex. These forces drive membrane molecules into nonequilibrium steady-state patterns such as nanoclusters. However, the molecular agents connecting membrane organization with cytoskeletal dynamics and stresses have remained unknown. Here, we show that two classes of ubiquitous ancestral nonmuscle myosins are deployed for the organization of different types of membrane components. Inner leaflet–localized class I myosins link outerleaflet glycosylphosphatidylinositol-anchored molecules to juxta-membrane actin filaments, whereas the more cortically localized Class II myosins operate on transmembrane proteins endowed with actin-binding capacity. Consistent with an active Flory-Huggins theory for phase separation, these observations show that the distinct motor-driven membrane molecules generate spatially segregated mesoscale domains, enriched in nanoclusters derived from different myosin classes. Moreover, chemically reversible posttranslational modifications such as palmitoylation enable concatenation of these domains by enhancing the affinity of the membrane domain constituents for each other. We anticipate that the segregation potential of the adenosine 5′-triphosphate (ATP)–fueled cell membrane is made available for the crucial purpose of modulating information transduction because it can be regulated in space and time during the construction of signaling cascades, underpinning functional plasma membrane organization.

## INTRODUCTION

The plasma membrane (PM) of every living cell is a two-dimensional fluid bilayer of lipids and proteins whose composition is regulated in a cell type–specific manner (*1*). Lipids and proteins are inhomogeneously distributed along the plane of the membrane and also asymmetrically distributed between the inner and outer leaflets of the bilayer (*2*–*5*). Its organization is crucial for its functioning as a dynamic interface for communication with the extracellular milieu and responding to internal states of the cell (*6*, *7*). While protein asymmetry is due to the presence of distinct peripheral proteins at the inner and outer leaflets of the PM, transverse lipid asymmetry is established by adenosine 5′-triphosphate (ATP)–driven flippases (*1*). The generation of lateral heterogeneity has been proposed to occur by interactions of membrane constituents as outlined in the fluid mosaic and the lipid raft models (*8*, *9*). However, it has been difficult to obtain evidence for thermodynamically driven phase segregation of membrane lipids at physiological temperatures (*10*–*12*). Instead, cross-linking of membrane protein (*11*, *13*) and actin-dependent clustering mechanisms (*14*, *15*) result in building membrane domains with distinct chemical compositions and properties (*11*, *16*).

On the basis of observations in living cells, it has been proposed (*14*) and validated in vitro (*17*) that alongside lipid phase separation or protein-protein, protein-lipid, or lipid-lipid interactions, actin filaments and motor activity create contractile flows culminating in dynamic platforms that promote transient nanoscale clustering of glycosylphosphatidylinositol (GPI)–anchored proteins (GPI-APs) as well as transmembrane (TM) proteins that interact with actin (*14*, *15*). These contractile platforms exhibit mesoscale organization, termed active emulsions (*16*). Evidence for these have primarily come from observations of the dynamics and distribution of nanoclusters of outer leaflet–localized GPI-APs, glycolipids, and inner leaflet lipid-anchored molecules (*15*, *18*). It is expected that the characteristics of these mesoscale domains depend on the specific molecules that are assembled in the contractile platforms. Nanoclustering of GPI-APs requires transbilayer interactions of long acyl chain–containing GPI anchors with lipid tails of inner leaflet–immobilized phosphatidylserine (PS) mediated by the enrichment of cholesterol (*19*). Because of these features, nanoclusters exhibit local lipid order (*lo*), well above the phase transition temperature (*T*_m_) of the membranes (*19*), and recruit other *lo*-preferring components. As a result, the mesoscale domains (active emulsions) of GPI-APs also exhibit *lo*-like characteristics (*16*). Although active emulsions of GPI-APs resemble phase-separated *lo* domains, the mechanism that generates these domains is distinct from equilibrium phase separation and is likely to have different properties. These features have propelled a new understanding of the PM as an “active composite” wherein the combined influence of the specific composition of the membrane and interactions with the underlying cytoskeleton controls its nano- and mesoscale organization (*16*, *17*).

Notwithstanding the progress made in understanding the active membrane composite (*3*, *20*), the precise molecular agents that drive this activity in the cell are still unknown. We provide evidence that the ancestral and ubiquitous class I and II nonmuscle myosin protein families (*21*) are the engines that power this organization. Class I and II myosins perform a diverse range of tasks in the cell. Class I myosins facilitate endocytosis, phagocytosis, and vesicle and protein transport on filamentous actin, including on protrusions such as microvilli and cilia, and initiate left-right asymmetry at the organismal scale because of their chiral character. The nonmuscle class II myosins are required for building contractile structures such as stress fibers and the actin cortex (*22*–*25*). Here, we find new functions for these two classes of myosin families, in the active and regulated organization of PM components, first by studying their distribution in the cell and then the consequence of their selective perturbation, consistent with a theoretical framework that emphasizes the role of active stresses in organizing membrane components.

In all metazoa, the actin cortex is infused with class II myosins, and here, we show that these motors are necessary for the construction of nanoclusters of membrane proteins that are associated with cortical actin. Simultaneously, we find that the ubiquitously present lipid-associated class I myosins drive the nano- and mesoscale arrangement of lipidic nanoclusters at the outer leaflet, likely due to their ability to associate with acidic lipid species at the inner leaflet of the PM (*19*). Thus, class I and II myosins serve as the main motors at the PM and in the actin cortex, driving the nanoscale clusters of membrane lipids and proteins, respectively. Last, we show that these motor-driven nanoclusters assemble into distinct domains with higher-order organization. They exhibit two kinds of active emulsions at the mesoscale with the capacity to sort or concatenate membrane components of different chemistries consistent with an active Flory-Huggins (AFH) theory, recently proposed by us (*26*). These domains are used in regulating the function of the membrane in many cellular processes, including in cell spreading.

### Class I and II myosins exhibit stratified distribution at the cortex

**Fig. 1.**
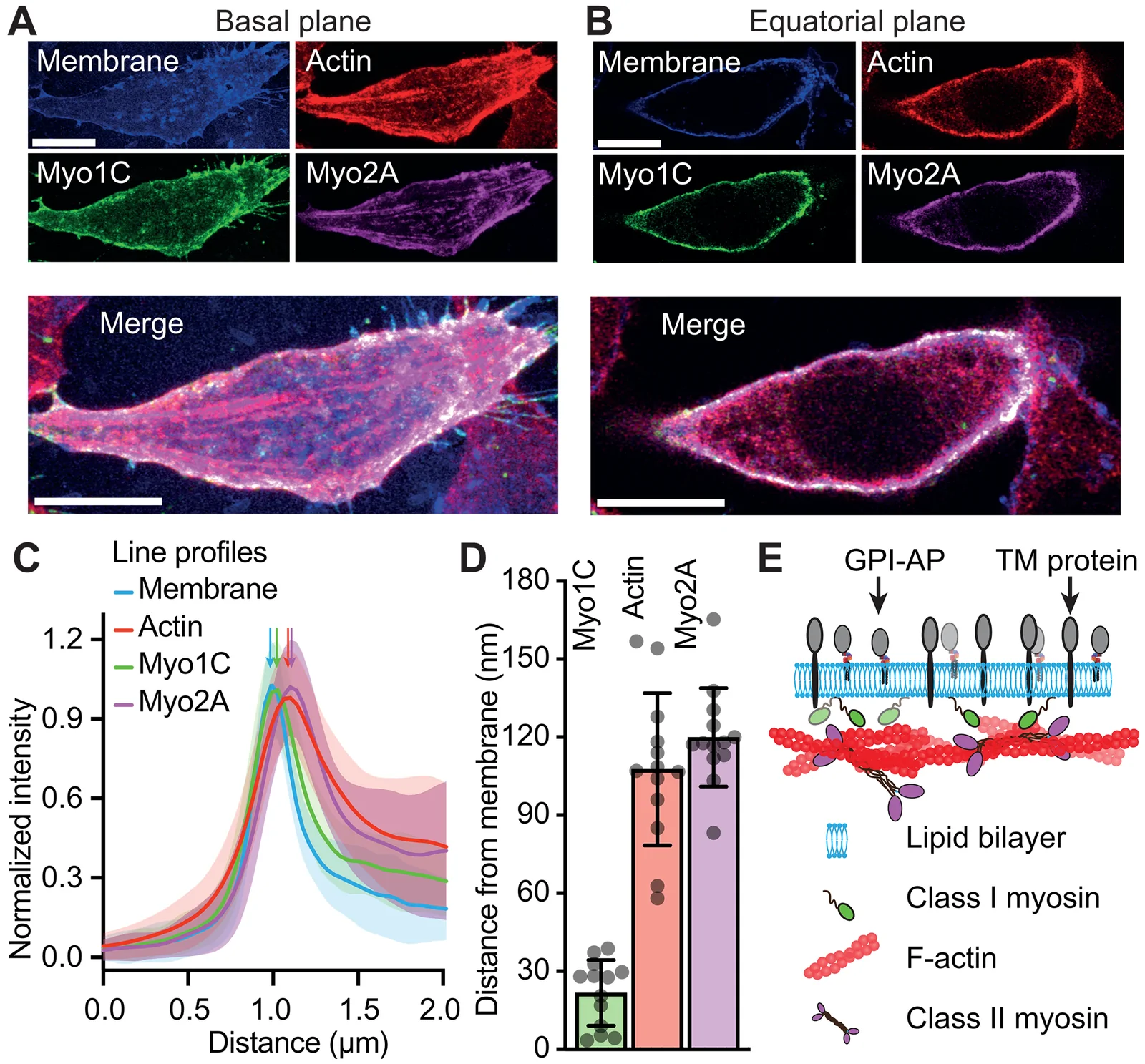
Distinct myosins decorate different layers of the cortical actin–supported PM. (**A** and **B**) Representative airy scan microscopy image of a fluorescently labeled membrane marker, Myo1C, F-actin, and Myo2A, in a single cell spread on fibronectin-coated glass, focused at the cell-glass interface (A) or away from the glass (B). (**C**) The plot shows the average of 69 membrane tangential line profiles (solid lines; shaded regions depict SD) taken from 13 cells, where the value of each line profile starting 1 μm outside and ending 1 μm into the cell was normalized to its maximum value. (**D**) Bar graphs show the average distance (±SD) of the myosins and actin from the PM determined from the position of the fitted maximum intensity of each protein with that of the PM marker. Each point corresponds to the mean distance of Myo1C, F-actin, and Myo2A from the PM obtained from the measurements within a single cell; when averaged across all cells, the distance corresponds to 21.7 ± 13, 107.6 ± 29, and 119.9 ± 19 nm, respectively. (**E**) The schematic shows the organization of GPI-APs at the outer leaflet of the PM, TM proteins inserted in the PM with class I myosin proteins at the inner membrane leaflet, and class II myosin entangled in the cortical F-actin meshwork. Scale bars, 10 μm.

Nonmuscle myosin class I motors have a lipid-binding domain (*27*), and class II motors are localized in the actin cortex (*28*), juxtaposed to the PM. Therefore, we examined the localization of these two classes of myosin motors with respect to the PM and the actin cortex in the same cell. For this, we imaged a representative of both class I [green fluorescent protein (GFP)-myosin 1C (Myo1C)] and class II myosins (mCherry-myosin 2A (Myo2A) together with PM and actin in Chinese hamster ovary (CHO) cells (Fig. 1). GFP-Myo1C colocalizes with the membrane marker in both the basal and equatorial planes, well separated from the major peak for actin (Fig. 1, A to C). mCherry-Myo2A, on the other hand, is predominantly localized within the membrane-proximal actin and is enriched on thick actin stress fibers (Fig. 1, A to C). Quantification of the distances involved shows the layering of the different proteins with respect to the membrane and actin cortex; class I myosin is distinctly membrane-associated (<30 nm away), whereas class II myosin is enriched between 100 and 120 nm in the juxta-membrane actin cortex (Fig. 1, C to E), consistent with the earlier reports (*28*, *29*).

### Class II myosins drive nanoclustering of TM actin-binding proteins

**Fig. 2.**
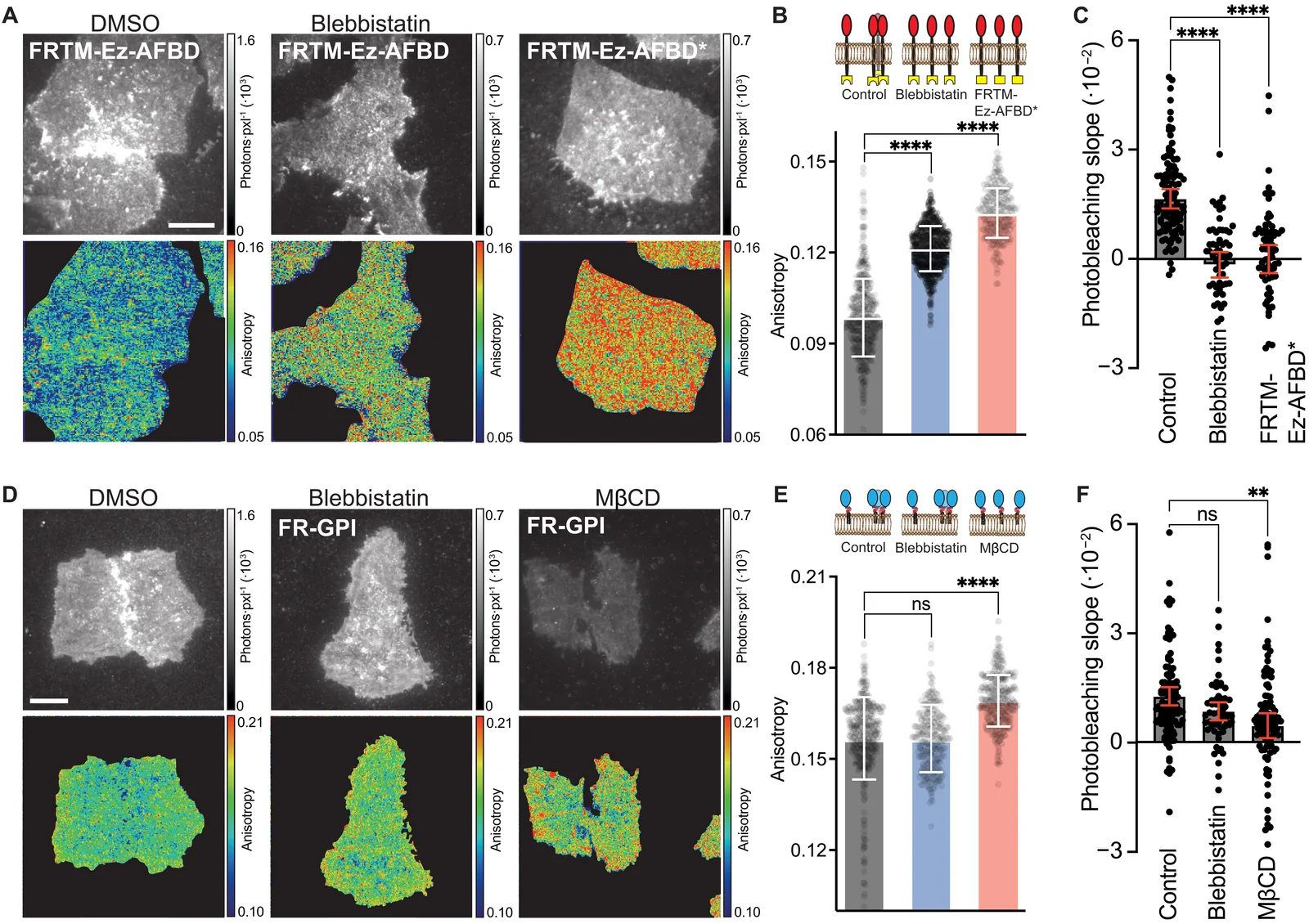
Effect of inhibition of class II myosin activity on membrane component organization. (**A**) Intensity (top) and anisotropy (bottom) images were obtained using TIRF microscopy from cells expressing a chimeric TM protein, FRTM-Ez-AFBD or FRTM-Ez-AFBD*, labeled with PLB and treated with vehicle (DMSO) or blebbistatin (100 μM) as indicated. (**B**) Bar graphs show the means (±SD) of emission anisotropy values (circles) collected from 4.5-μm^2^ ROIs obtained from emission anisotropy images collected as in (A). Data for the control, blebbistatin, and FRTM-Ez-AFBD* were obtained from 40 cells (446 ROIs), 51 cells (609 ROIs), and 13 cells (239 ROIs), respectively. (**C**) Bar graphs show the means (±SD) of photodilution slopes of emission anisotropy from chimeric proteins following perturbations as in (A). Data for the control, blebbistatin, and FRTM-Ez-AFBD* were obtained from 100 cells (*n* = 7), 61 cells (*n* = 4), and 70 cells (*n* = 5), respectively. (**D**) Intensity (top) and anisotropy (bottom) images obtained using TIRF microscopy from cells expressing FR-GPI and labeled with PLB and treated as indicated. (**E**) Bar graphs show the means (±SD) of emission anisotropy values (circles) collected from 4.5-μm^2^ ROIs obtained from emission anisotropy images collected as in (D). Data were obtained from 32 cells (396 ROIs), 19 cells (270 ROIs), and 25 cells (312 ROIs) for the control, blebbistatin, and MβCD experiments, respectively. (**F**) Bar graphs show the means (±SD) of photodilution slopes of emission anisotropy from FR-GPI following perturbations as in (E). Data were obtained from 109 cells (*n* = 6), 55 cells (*n* = 4), and 106 cells (*n* = 6) for the control, blebbistatin, and MβCD experiments, respectively. LUT, gray scale, total photons detected; color, anisotropy values. Scale bars, 10 μm. ****, **, and ns correspond to *P* < 10^−4^, *P* < 10^−2^, and *P* > 0.05, respectively.

The actin cortex forms a dense network juxtaposed to the PM. Many membrane proteins associate with cortical actin filaments. Class II myosin motors can generate local contractile stresses on the actin filaments and, as a result, cluster the actin-associated proteins at the nanoscale (*14*, *30*, *31*). Therefore, we tested whether inhibiting class II myosin activity would influence the nanoclustering of a TM protein with cytoplasmic actin-binding capacity. We expressed a chimeric TM protein, FRTM-Ez-AFBD, where the extracellular domain of the folate receptor (FR) coupled to the TM region of the immunoglobulin G receptor (TM) is linked to the cytoplasmic actin filament–binding domain from Ezrin (Ez-AFBD) in cells [FRTM-Ez-AFBD cells; (*14*)]. The FR domain may be labeled with a fluorescent analog of folate [pteroyl-lysyl-BODIPY (PLB); (*15*)]. Labeled FRTM-Ez-AFBD exhibits homo–Förster resonance energy transfer (FRET) as a result of nanoclustering, as measured by reduced fluorescence anisotropy compared to the actin-binding mutant of FRTM-Ez-AFBD (Arg579-> Ala or R579A in FRTM-Ez-AFBD*) (*14*, *32*). FRTM-Ez-AFBD exhibited an increase in anisotropy when class II myosin activity was inhibited with blebbistatin, a compound that interferes with myosin power stroke and reduces its ability to bind actin (Fig. 2, A and B) (*33*). This is consistent with a loss in nanoclustering and was independently confirmed by monitoring the change in emission anisotropy upon photobleaching of labeled proteins. When fluorescently labeled proteins are present in nanoclusters, photobleaching reduces the number of active fluorophores, resulting in a decrease in homo-FRET and a corresponding increase in anisotropy (positive slope). By contrast, anisotropy of unclustered proteins is expected to remain unchanged (null slope) upon photobleaching (see the schematic in fig. S1A) (*34*). The positive slope observed in untreated cells with photobleaching of FRTM-Ez-AFBD signifies nanoclustering in contrast to the null slope obtained in blebbistatin-treated cells (Fig. 2C). The null slope is similar to that obtained for the actin-binding mutant protein, FRTM-Ez-AFBD* (Fig. 2C and fig. S1), consistent with the loss of nanoclustering. In addition, treatment of the cells with a cocktail of inhibitors, MLY [ML7 + Y27632; against the regulatory light chain phosphorylating kinases ROCK (Rho-associated protein kinase) and MLCK (myosin light chain kinase)] (*35*), also resulted in a loss of FRTM-Ez-AFBD clustering (fig. S1E). Consistent with these data obtained with the synthetic actin-binding FRTM-Ez-AFBD, we have found that nanoscale clustering of various naturally occurring TM proteins is also mediated by class II myosin activity (*30*, *31*). In particular, actin-dependent nanoclustering of cell-matrix adhesion protein CD44 and cell-cell adhesion protein E-cadherin in *Drosophila* at the free cell surface is perturbed by MLCK and class II myosin inactivation (*30*, *31*). Here, the association of CD44 and E-cadherin with actin is mediated by the cytosolic proteins, ezrin and α-catenin, respectively, that link filamentous actin to the cytoplasmic tails of these proteins.

We next addressed whether class II myosin activity also influenced the actin-driven organization of lipids in the membrane by examining the nanoclustering of GPI-APs, a representative of long-acyl-chain lipids at the outer leaflet (*19*, *32*, *36*). In stark contrast to the TM actin-binding proteins, when cells expressing GPI-anchored FR (FR-GPI) were treated with blebbistatin, there was minimal loss of clustering of the lipid-anchored protein as noted by the absence of any significant change in anisotropy or photobleaching slope of PLB-labeled FR-GPI (Fig. 2, D to F). In comparison, depletion of cholesterol—a crucial component of the GPI-AP nanoclusters—from the membrane resulted in a significant increase in FR-GPI anisotropy and decrease in photobleaching slope, as reported earlier (*32*). In *Drosophila* hemocytes, removal of class II myosin heavy chain (zip) or regulatory light chain (sqh) by UAS-GAL4–driven RNA interference (RNAi) also did not result in a loss of GFP-GPI clustering (fig. S2, A and B), whereas the actin-dependent clustering of the TM *Drosophila* E-cadherin is lost (*31*). These results suggest that while nanoscale clustering of membrane proteins that are able to bind actin is mediated by class II myosins, outer-leaflet lipid-anchored molecules are likely to be clustered by lateral stresses derived from different molecular agents.

### Class I myosins drive nanoclustering of GPI-APs

**Fig. 3.**
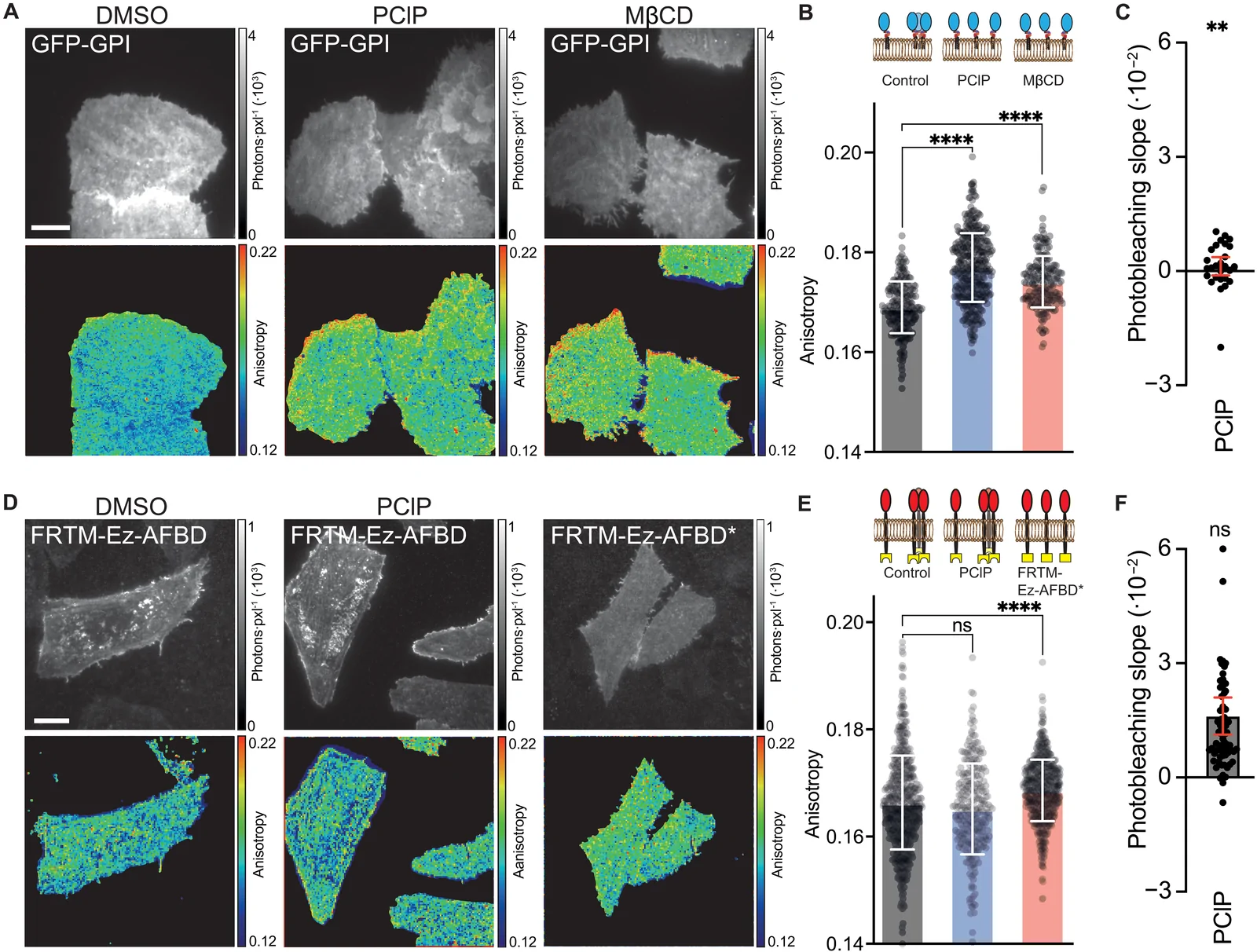
Effect of class I myosin inhibition on membrane component organization. (**A**) Intensity (top) and anisotropy (bottom) images obtained using TIRF microscopy from cells expressing GFP-GPI and treated with the vehicle (DMSO), myosin 1 inhibitor (PClP at 5 μM), or MβCD (10 mM). (**B**) Bar graphs show the means (±SD) of emission anisotropy values (circles) collected from 4.5-μm^2^ ROIs obtained from emission anisotropy images collected as in (A). Data were obtained from 48 cells (218 ROIs), 49 cells (415 ROIs), and 19 cells (205 ROIs) for the control, PClP, and MβCD experiments, respectively. (**C**) The bar graph shows the means (±SD) of the photodilution slopes of emission anisotropy from FR-GPI labeled with PLB, following PClP treatment from 27 cells (*n* = 3); the *P* value comparison is made with the control experiment in Fig. 2F. (**D**) Intensity (top) and anisotropy (bottom) images were obtained using TIRF microscopy from cells expressing chimeric TM actin-binding protein FRTM-Ez-AFBD or FRTM-Ez-AFBD* labeled with PLB, and FRTM-Ez-AFBD–expressing cells were treated with the vehicle (DMSO) or PClP (5 μM). (**E**) Bar graphs show the means (±SD) of emission anisotropy values (circles) collected as above from images as in (D). Data for the control, PClP, and FRTM-Ez-AFBD* were obtained from 50 cells (620 ROIs), 49 cells (299 ROIs), and 53 cells (683 ROIs), respectively. (**F**) The bar graph shows the means (±SD) of the photodilution slopes of emission anisotropy from PLB-labeled FRTM-Ez-AFBD from 68 cells (*n* = 3) after treatment with PClP. The *P* value comparison is made with the control experiment in Fig. 2C. LUT, gray scale, total photons detected; color, anisotropy values. Scale bars, 10 μm. ****, **, and ns correspond to *P* < 10^−4^, *P* < 10^−2^, and *P* > 0.05, respectively.

The presence of the lipid-binding and evolutionarily conserved class I myosin isoforms juxtaposed to the cell membrane (Fig. 1 and fig. S3, A to C) raises the possibility that these proteins may be involved in the formation of lipidic clusters independent of class II myosin function. There are eight human myosin 1 isoforms (myosins 1A, 1B, …, 1H). Each isoform is a single heavy chain comprising of an actin-binding motor head domain, a calmodulin-binding neck region, and a lipid-binding tail domain (fig. S3, A to C) (*27*). We used the small-molecule inhibitor pentachloropseudilin (PClP) that decreases the interaction of class I myosin with cortical actin to inhibit class I myosin activity across all isoforms (*37*, *38*). When CHO cells expressing fluorescently labeled GPI-APs (FR-GPI or GFP-GPI cells) were treated with PClP, there was an increase in the anisotropy of GFP-GPI (Fig. 3, A and B) and PLB-labeled FR-GPI (fig. S2C) compared to the vehicle-treated control. This increase in anisotropy was comparable to that measured upon depleting cholesterol from the membrane. Photobleaching of fluorophores confirmed that inhibition of class I myosin motor activity resulted in loss of homo-FRET, consistent with a loss of nanoclustering of GPI-APs (Fig. 3C). By contrast, when FRTM-Ez-AFBD cells were treated with PClP, nanoclustering of the TM protein was unaffected (Fig. 3, D to F). These results were not cell type–specific; PClP treatment also abrogated GPI-AP nanoclustering in human origin U2OS and AGS cells (fig. S2C).

Class I myosin isoforms are expected to bind lipids via their tail domains and have a broad expression range across tissues. Some isoforms such as MYO1E and MYO1F also bind proteins, and others, including MYO1A, MYO1G, and MYO1H, are expressed only in specialized cell types and tissues (*27*, *39*, *40*). To identify which specific class I myosin isoform(s) may influence the general mechanism of GPI-AP clustering by connecting membrane lipids to the actin cytoskeleton, we examined the role of three of the lipid-binding, PM-localized isoforms that are widely expressed: MYO1B, MYO1C, and MYO1D. In GFP-GPI–expressing U2OS cells, an increase in anisotropy of GFP-GPI was only observed if the levels of MYO1B, MYO1C, and MYO1D were all simultaneously reduced by small interfering RNA (siRNA) targeting all these human origin isoforms (fig. S2, D to H). This conclusively implicates class I myosins in lipid-based (GPI-AP) nanoclustering. This was further corroborated by UAS-GAL4–driven RNAi depletion of the orthologous Myo1C/H (Myo61F) and Myo1D/G (Myo31DF) in *Drosophila* hemocytes (fig. S2, A and B).

### Class I myosins link cytoplasmic actin with outerleaflet GPI-APs via PS

**Fig. 4.**
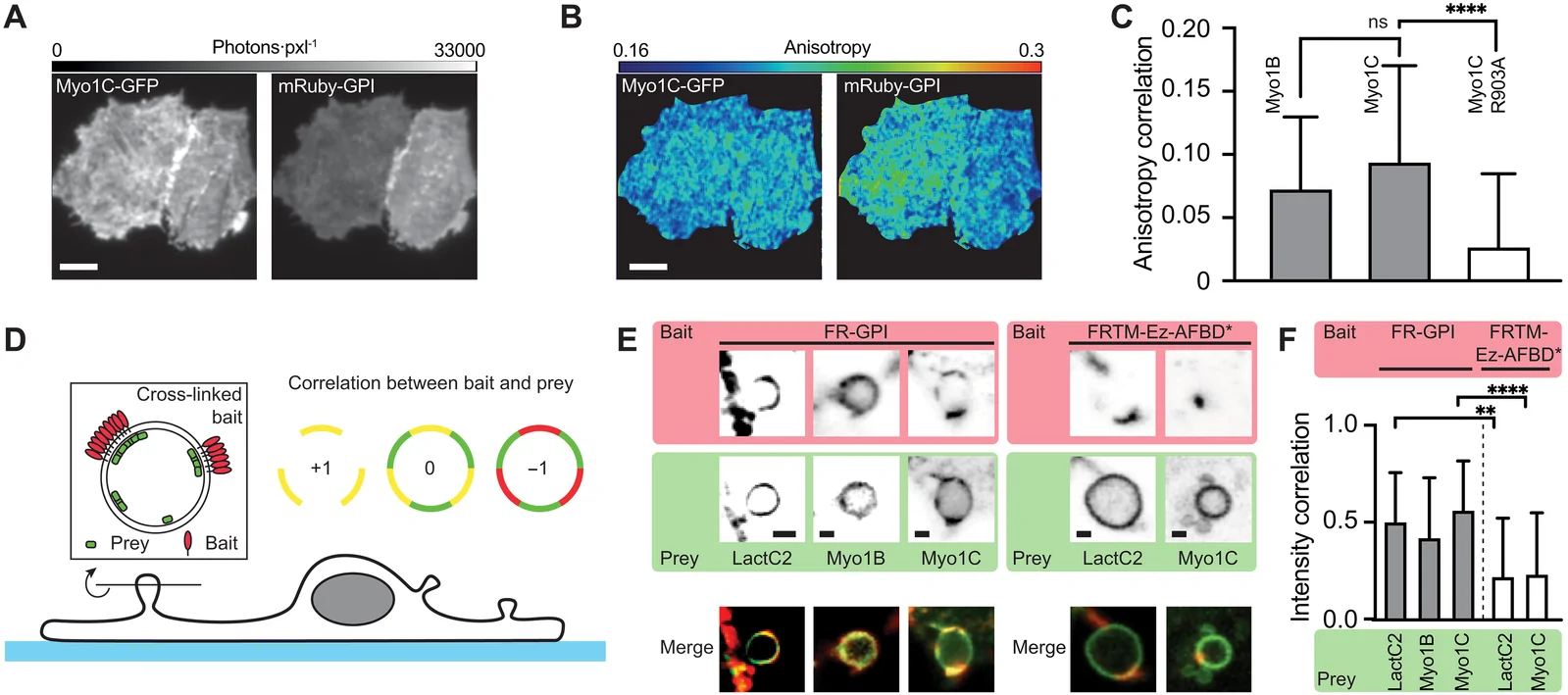
Class I myosins correlate with GPI-APs. (**A** and **B**) Intensity (A) and anisotropy (B) images of cells coexpressing mRuby-GPI and Myo1C-GFP. (**C**) Dual-color anisotropy (Pearson’s) cross-correlation coefficient from cells coexpressing both mRuby-GPI and Myo1B-GFP (46 cells), Myo1C-GFP (114 cells), or Myo1C(R903A)-GFP (58 cells). The graph shows quantification of correlation coefficients where the bars represent the means and SD of data derived from the indicated number of cells. (**D**) Schematic representation of the assay to test the colocalization of outerleaflet proteins (bait) and inner-leaflet (prey) proteins in cell-attached blebs. (**E**) Representative confocal images of the equator plane of cell-attached blebs with either FR-GPI or FRTM-Ez-AFBD* as cross-linked bait and LactC2-GFP (PS-lipid–binding protein), GFP-Myo1B, or GFP-Myo1C as prey proteins at the inner leaflet. (**F**) The graph shows quantification of the bait-prey intensity correlation index of the proteins visualized in (E) from multiple cell-attached blebs, where the bars represent the means and SD derived from the indicated number of blebs [FR-GPI blebs with LactC2 (80), Myo1B (20), and Myo1C (84) and FRTM-Ez-AFBD* blebs with LactC2 (20) and Myo1C (50)]. Scale bars, 10 μm (A and B) and 2 μm (E). ****, **, and ns correspond to *P* < 10^−4^, *P* < 10^−2^, and *P* > 0.05, respectively.

If class I myosins are involved in clustering GPI-APs and the activity of these myosins results in their clustering, we predict that nanoclustered GPI-APs would be spatially correlated across the bilayer with corresponding clustered myosin 1 isoforms. To test this, we coexpressed GFP-tagged Myo1B or Myo1C isoforms with mRuby-GPI in cells and assessed the global covariance between myosin and GPI-AP clustering using a two-color anisotropy correlation analysis (*16*). Our results show that the GPI-AP nanoclusters at the outer leaflet are correlated with cluster-rich regions of Myo1B and Myo1C at the inner leaflet. In contrast, when we expressed a mutant form of Myo1C (R903A) that reduces the association of myosin with negatively charged lipid species (*29*), the correlation is abrogated (Fig. 4, A to C).

We previously demonstrated that transbilayer coupling between long acyl chaincontaining GPI-APs at the outer leaflet and long acyl chain PS in the inner leaflet is necessary for their clustering. Because class I myosins bind actin via their motor domain and anionic lipids [largely phosphatidylinositol 4,5-bisphosphate (PIP_2_) and PS] via their tail domain, they are well positioned to couple the actin cytoskeleton at the inner leaflet to outerleaflet GPI-APs (*29*, *41*). To test this, we generated cell-attached blebs in FR-GPI–expressing cells and cross-linked the GPI-AP with antibodies to produce large, optically resolved membrane patches (*19*) that serve as “bait” regions to recruit interacting molecules at the inner leaflet (see the schematic in Fig. 4D). As expected, GPI-AP cross-linking recruited PS lipids, detected by the PS sensor LactC2-GFP (Fig. 4, E and F), consistent with a previous study (*19*). GFP-Myo1B and GFP-Myo1C were also enriched at these patches (Fig. 4, E and F). In contrast, cross-linking the TM protein FRTM-Ez-AFBD* failed to recruit GFP-Myo1C (Fig. 4, E and F). We also found no relationship between “bait” clustering strength and “prey” recruitment. These results indicate that class I myosins are preferentially recruited to GPI-AP and PS-enriched membrane domains, supporting their role as active linkers that couple cytoplasmic actin to PS at the inner leaflet and, through transbilayer acyl chain interactions, to GPI-APs and glycolipids at the outer leaflet, thereby behaving as “active linkers.”

**Fig. 5.**
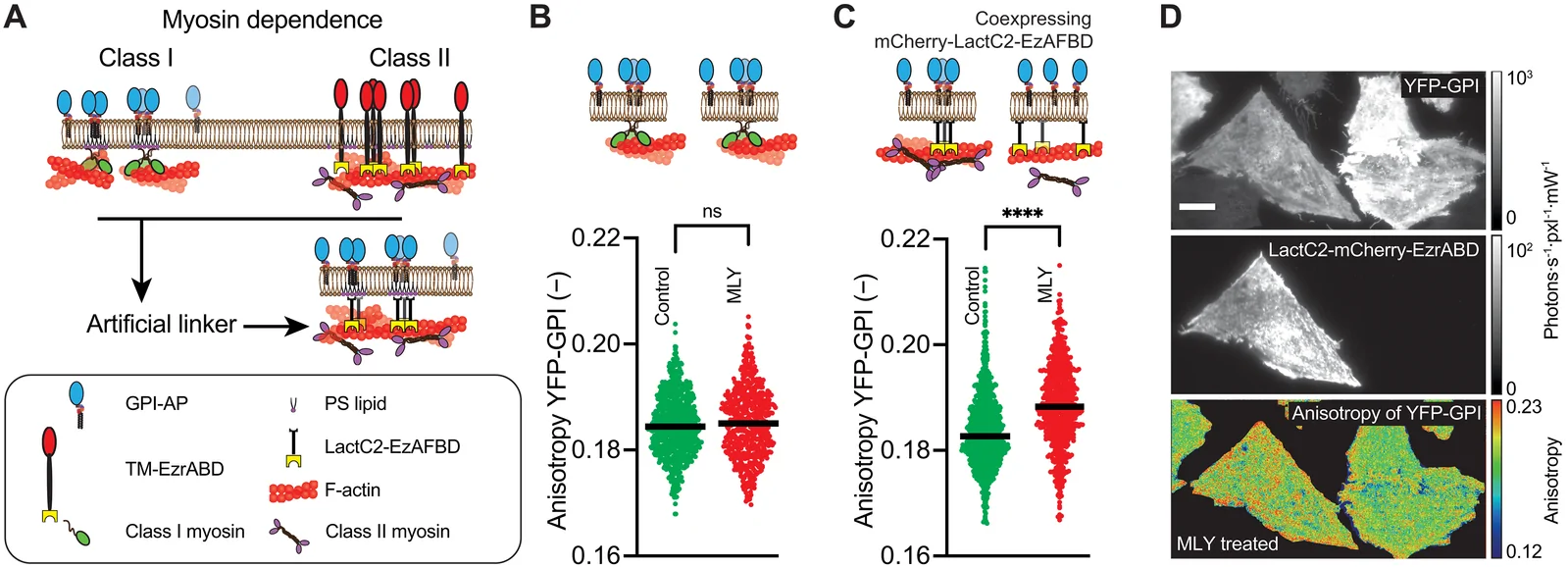
Linker molecules determine myosin dependence. (**A**) Model for the bifurcation of the roles of class I and II myosin motors on association with membrane lipids and proteins, respectively generating distinct nanoscale clusters. Note that the synthetic construct containing both PS binding and actin binding would confer the ability of myosin 2 to promote membrane lipid reorganization. (**B** and **C**) Graphs showing the differential sensitivity of YFP-GPI clustering to a myosin class II inhibitor cocktail, MLY (20 μM ML7 and Y27632), in the absence or presence of the synthetic PS- and actin-binding linker LacC2-EzAFBD. Data were obtained from 77 cells (1081 ROIs) and 105 cells (1041 ROIs) in (B) and 54 cells (1108 ROIs) and 53 cells (649 ROIs) in (C) for the control and MLY perturbations, respectively. (**D**) Intensity (top and middle) and anisotropy (bottom) images of YFP-GPI cells coexpressing mCherry-LacC2-EzAFBD (middle) exhibiting sensitivity to the MLY cocktail. Scale bar, 10 μm (D). **** and ns correspond to *P* < 10^−4^ and *P* > 0.05, respectively.

We next asked whether we could redirect GPI-APs to interact with the class II myosin–driven clustering mechanism by linking PS directly to an actin-binding domain. To this end, we used a synthetic PS-actin linker consisting of LactC2 and Ez-AFBD (LactC2-EzAFBD) (see the schematic in Fig. 5A). CHO cells expressing yellow fluorescent protein (YFP)-GPI were transiently transfected with mCherry-LactC2-EzAFBD, and the emission anisotropy of YFP-GPI in transfected and untransfected cells was compared for its sensitivity to class II myosin activity by treating with an inhibitor cocktail against myosin light chain phosphorylation: MLY (ML7 and Y27632) (Fig. 5, B and C, and fig. S4). Consistent with a redirection of the clustering mechanism, we observed that the anisotropy of YFP-GPI developed sensitivity to inhibition of class II myosin activity only in cells coexpressing LactC2-EzAFBD (Fig. 5, B to D).

Class I myosins have a plekstrin homology domain in their tail domain that binds PIP_2_, while additional positively charged residues in its tail may facilitate association with other negatively charged lipids such as PS (*29*). Although binding to PIP_2_ was demonstrated before (*29*), how class I myosins interact with PS is less well understood. We deployed AlphaFold 3 (*42*) and the CCD2MD lipid library (*43*) to co-fold the tail domains of Myo1B and Myo1C in the presence of anionic lipid molecules [PIP_2_ and DOPS (dioleylphosphatidylserine); 1:10], approximating their relative abundance in the PM (fig. S3D). We find that both Myo1B and Myo1C have overlapping and distinct residues that can associate with PIP_2_ and PS. While PIP_2_ occupies a high-affinity binding pocket as reported, additional dispersed basic patches of both myosins can still support electrostatic interactions with anionic lipids such as PS (fig. S3, C to G).

### Spatial relationships of the nanocluster-rich domains of GPI-APs and TM actin-binding proteins

**Fig. 6.**
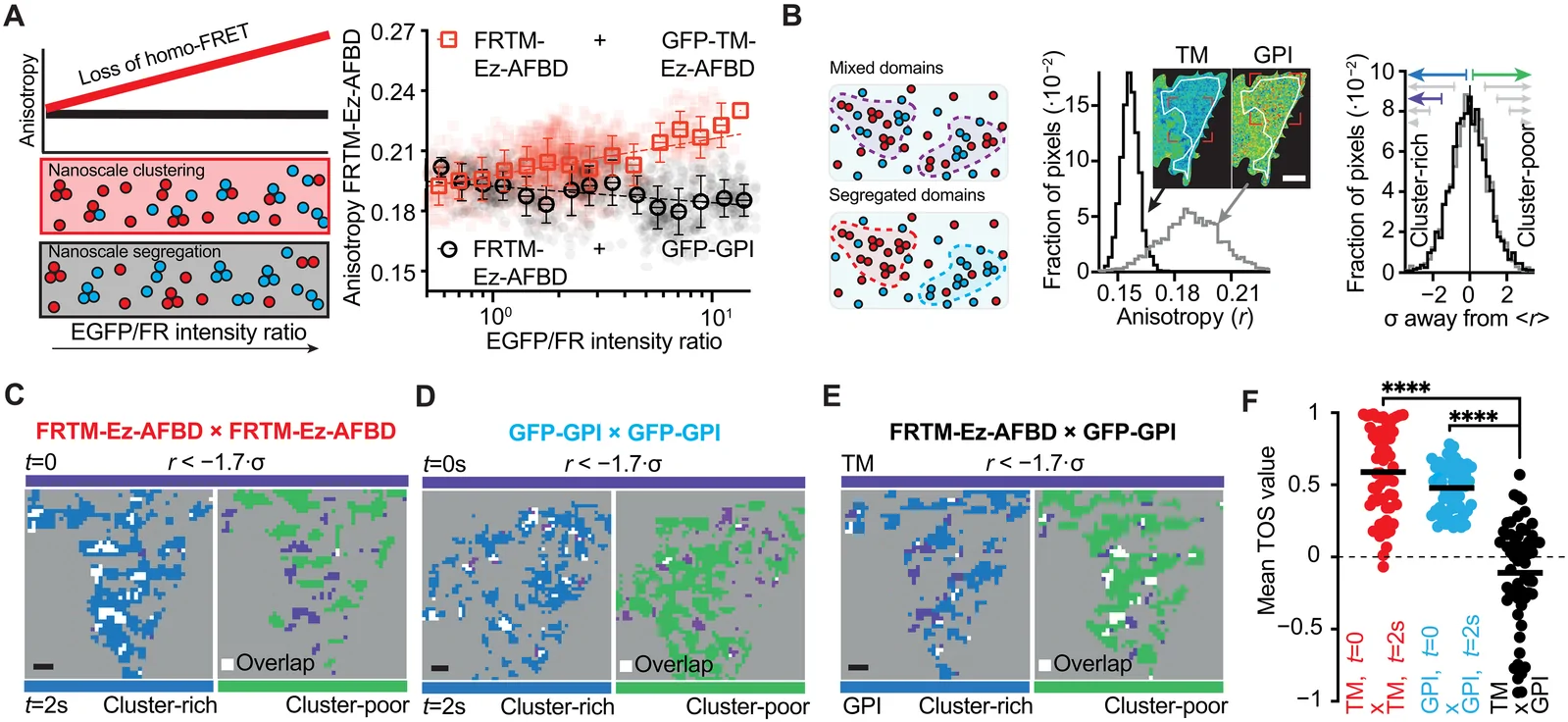
Spatial sorting of myosin activity zones. (**A**) Left: Schematic illustrating how the extent of nanoscale cohabitation influences fluorescence anisotropy measurements as a function of the relative expression of GFP- and PLB-labeled proteins. Right: Change in anisotropy as a function of the ratio of GFP to PLB-labeled FRTM-Ez-AFBD in cells coexpressing both proteins. Note that anisotropy increases when GFP-TM-Ez-AFBD is coexpressed with FRTM-Ez-AFBD; red (1421 ROIs from 67 cells, *n* = 2), square data points were obtained by averaging the emission anisotropy of FRTM-Ez-AFBD at the indicated GFP/FR ratio bin (±SD). However, there is no change when GFP-GPI is coexpressed with FRTM-Ez-AFBD; black (1021 ROIs from 59 cells, *n* = 2), open circles are the means ± SD of anisotropy at the different ratio bins. (**B**) Schematic of possible mesoscale cluster arrangements (left) and histograms of the raw (middle) and normalized (right) pixel anisotropy values of the same selected cell (see anisotropy image insets) for FRTM-Ez-AFBD (black line) and the GFP-GPI (gray line) signals. (**C** to **E**) Threshold images of pixels corresponding to strongly cluster-rich pixels (<−1.7·σ, purple pseudocolored) in one image together with the weakly cluster-rich (<〈r〉, blue pseudocolored) or weakly cluster-poor pixels (>〈r〉, green pseudocolored) of the corresponding second image: temporal FRTM-Ez-AFBD correlation (C), temporal GFP-GPI correlation (D), and FRTM-Ez-AFBD with GFP-GPI cross-correlation (E). Note the abundance of overlapping pixels (white) between strongly and weakly cluster-rich regions [(C) and (D), left panels] and the lack of overlap with weakly cluster-poor regions (C and D, right panels) for the temporal correlations of the same component. Such overlap is absent when displaying the strongly versus weakly cluster-rich thresholded images of FRTM-Ez-AFBD and GFP-GPI (E, left panel). The amount of pixel overlap can be contrasted to chance overlap and quantified as a TOS value (see Materials and Methods for details). (**F**) Graph of the mean TOS value between FRTM-Ez-AFBD and GFP-GPI (58 cells, *n* = 2). Their averaged temporal and cross-correlation TOS values have been obtained from the area enclosed by the dashed line in the TOS matrix of fig. S5E. LUT, color, anisotropy values. Scale bars, 10 μm [images in (B)] and 2 μm [pixels maps in (C) to (E)]. **** corresponds to *P* < 10^−4^.

Our results thus far provide evidence for at least two types of active clustering pathways in the membrane: one localized at the membrane where class I myosin drives the nanoclustering of lipid-linked membrane components and another mediated by the more cortically localized class II myosins that generate nanoscale clusters of TM proteins linked to actin. Our earlier work showed how nanoscale clustering of membrane components was a natural consequence of the contractile stresses applied by actomyosin (*14*). Given that the molecular agencies for these contractile stresses are in different strata of the cortex, we asked whether there is a segregation at the nanoscale. We rationalized that when two proteins, e.g., A and B, are part of the same nanocluster, A will increasingly dilute out B within the nanocluster as a function of increasing concentration of A. When A and B are tagged to different fluorophores, dilution of B by A can be directly read out by an increase in anisotropy of B because of increased intermolecular distance and decreased homo-FRET as increasing amounts of A displace B from the nanoclusters (Fig. 6A). To obtain evidence for coclustering or segregation at the nanoscale, we coexpressed the following combinations of FR- and GFP-linked (i) TM-Ez-AFBD (FRTM-Ez-AFBD and GFP-TM-Ez-AFBD) or (ii) GPI anchors (- and GFP-GPI) or (iii) both (FRTM-Ez-AFBD and GFP-GPI). In cells expressing both FRTM-Ez-AFBD and GFP-TM-Ez-AFBD, the anisotropy value of FRTM-Ez-AFBD increased as a function of the expression level of GFP-TM-Ez-AFBD (Fig. 6A, red data and line in graph). This is possible if GFP-TM-Ez-AFBD stochastically “diluted” FRTM-Ez-AFBD in the nanoclusters. Similarly, when FR-GPI and GFP-GPI were coexpressed, the extent of homo-FRET of either of the two was inversely proportional to the relative expression level of the other, consistent with coclustering of the two proteins at the nanoscale (*32*). By contrast, such dilution of FRTM-Ez-AFBD clusters by GFP-GPI was not observed, indicating the lack of coclustering of these distinct proteins (Fig. 6A, black data and line in graph). This shows that at the cell surface, the diverse myosin machineries build distinct nanoscale clusters.

In earlier studies, we showed that nanoclusters tend to come together and enrich in mesoscale regions with a characteristic length scale of about ∼450 nm, termed as active emulsions (*14*, *16*). To assess the extent of overlap between nanocluster-rich mesoscale domains of FRTM-Ez-AFBD and GFP-GPI, we used a metric called threshold overlap scores (TOSs). This metric is particularly well suited for assessing colocalization of spatial features because thresholding highlights salient image features while minimizing noise, and the resulting overlap is normalized to that expected by chance (see Methods) (*44*). To measure TOS, we acquired near-simultaneous fluorescence emission anisotropy images from cells coexpressing PLB-labeled FRTM-Ez-AFBD and GFP-GPI in separate fluorescence channels. Pixels in each region of interest (ROI) were classified according to the extent of their deviation from the mean anisotropy of the region using thresholds defined by successive SD intervals above and below the mean (Fig. 6B). This generated graded categories ranging from cluster-rich (low anisotropy) to cluster-poor (high anisotropy) pixels for each channel (Fig. 6, C to E, and fig. S5, A to C). TOS was subsequently used to quantify the nonrandom spatial overlap between corresponding categories after correcting for the overlap expected by chance (fig. S5D). We focused our analysis on the overlap between cluster-rich pixels, corresponding to mesoscale emulsions (fig. S5E). In living cells, this metric revealed significant temporal persistence of cluster-rich mesoscale emulsions, with images acquired 2 s apart exhibiting substantial self-overlap for both FRTM-Ez-AFBD and GFP-GPI (Fig. 6, C, D, and F, and fig. S5E). Across multiple cells, this was reflected in high mean TOS values (TOS_FRTM-EzAFBD_ = 0.59 ± 0.31 and TOS_GFP-GPI_ = 0.48 ± 0.16; Fig. 6F), indicating that each probe forms a stable network of cluster-rich mesoscale domains. In contrast, comparing GFP-GPI and FRTM-Ez-AFBD within the same cells yielded a slightly negative overlap score (TOS = −0.11 ± 0.37; Fig. 6, E and F). These results are consistent with minimal overlap between the mesoscale regions generated by the distinct myosin motors, as illustrated schematically in Fig. 6B.

### AFH theory predicts minimal overlap of domains generated by active stresses with tunable concatenation

**Fig. 7.**
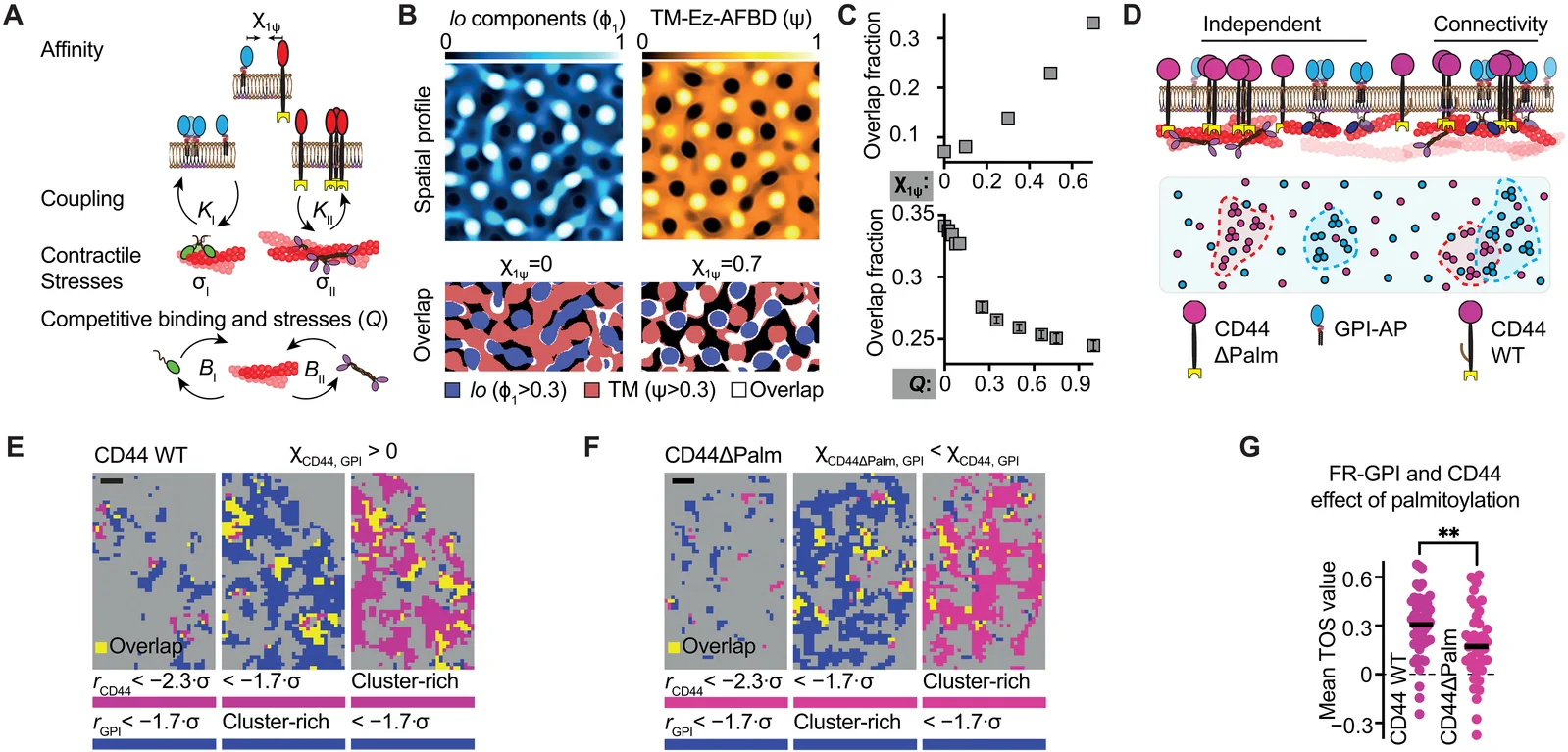
AFH theory and concatenation of myosin activity zones. (**A**) Elements of the components for the AFH theory incorporating two agents of contractile stresses ($\sigma_{I,\text{II}}$). The theory can be separated into (i) an actomyosin component where competitive myosin binding and stresses (***Q***) determine the local accumulation of active class I or class II components and (ii) in-plane interactions strength $\left(\chi_{1\psi }\right)$ between the different membrane components coupled to distinct stress agents (see also the Supplementary Materials). (**B**) Representative area-fraction images resulting from numerical simulations of the AFH theory for the *lo*-phase (top left) and TM-Ez-AFBD (top right). Bottom row: Two representative overlap images for mesoscale domains (area fraction >0.3) where the interaction parameters $\left(\chi_{1\psi }\right)$ between the *lo*-phase and TM-Ez-AFBD were set at 0 (left) and 0.7 (right). (**C**) Fraction overlap as a function of interaction parameter (top) or competitive stresses between the two classes of myosin (bottom). (**D**) Cartoon displaying the possibility for connectivity leading to concatenation of mesoscale domains organized by the different classes of myosin motors. WT, wild type. (**E** and **F**) Thresholded images of a region of a cell coexpressing FR-GPI with CD44 WT or CD44ΔPalm showing pixels corresponding to strongly cluster-rich domains (CD44: <−2.3·σ or −1.7·σ, purple pseudocolored; GPI: <−1.7·σ, blue pseudocolored) and weakly cluster-rich domains (CD44: <〈r〉, purple pseudocolored; GPI: <〈r〉, blue pseudocolored). Note that while weakly cluster-rich regions of both CD44 constructs display some overlap (yellow pixels) with weakly cluster-rich regions of FR-GPI, however, only strongly cluster-rich CD44 WT regions are significantly concatenated with strongly cluster-rich FR-GPI regions (E and F, left panels). (**G**) Graph of the mean TOS values between CD44 and FR-GPI with and without the palmitoylation sites. LUT, color, anisotropy values. Scale bars, 2 μm [pixels maps in (E) and (F)]. ** corresponds to *P* < 10^−2^.

To understand the basis for the mesoscale patterning of membrane components driven by multiple membrane-localized active stresses, we elaborated an AFH theory, originally built to describe these active emulsions built by a single contractile agent (*26*). Here, we extend this analysis to incorporate two agents of contractility, namely class I and II myosins that drive the clustering of class I myosin–binding *lo* components and the class II myosin–associated actin-binding component FRTM-Ez-AFBD, respectively. For simplicity, we formulate the AFH theory in terms of the dynamics of the area fractions of the *lo*-preferring component (GPI-AP) and TM-Ez-AFBD on the membrane in juxtaposition with the actomyosin cortex. The dynamics is driven by forces that arise from (i) passive interactions between the components and (ii) active stresses on the *lo* component and TM-Ez-AFBD arising from class I and II myosins, respectively. In turn, active stresses depend on the local concentrations and contractile flows of class I and II myosins in the cortex as described by active hydrodynamics equations (*26*), which incorporate their differential turnover, competitive binding, and contractile activity (see the schematic in Fig. 7A, the Supplementary Materials, and fig. S6). The theory predicts that the *lo* component segregates from the bulk lipid disordered *ld* phase even at temperatures (*T*) higher than the critical temperature (*T*_c_) or *T* > *T*_c_, as verified in earlier experiments (*16*), driven by contractile flows associated with the myosin activity linked to the *lo* component. In addition, contractile flows associated with class II myosin drive TM-Ez-AFBD into forming distinct domains (Fig. 7B and fig. S6). Mesoscale domains of each component, defined for the numerical simulations as regions with area fractions above a threshold (0.3), show minimal overlap where the *lo* component and TM-Ez-AFBD do not interact (Fig. 7B), consistent with experiments (Fig. 6, B to F). Unlike usual phase segregation, these mesoscale domains are not homogeneous but show granularity, with smaller regions of high density, identified with nanoclusters. The theory predicts that the extent of overlap (concatenation) at the mesoscale depends on competitive binding and contractile activity of the myosins through the parameter *Q* (Fig. 7C, top panel; see also the Supplementary Materials). Moreover, the theory predicts that the extent of concatenation of the distinct mesoscale regions can be enhanced by increasing the in-plane affinity between their components (Fig. 7, B and C, bottom panel, and fig. S6F).

**Fig. 8.**
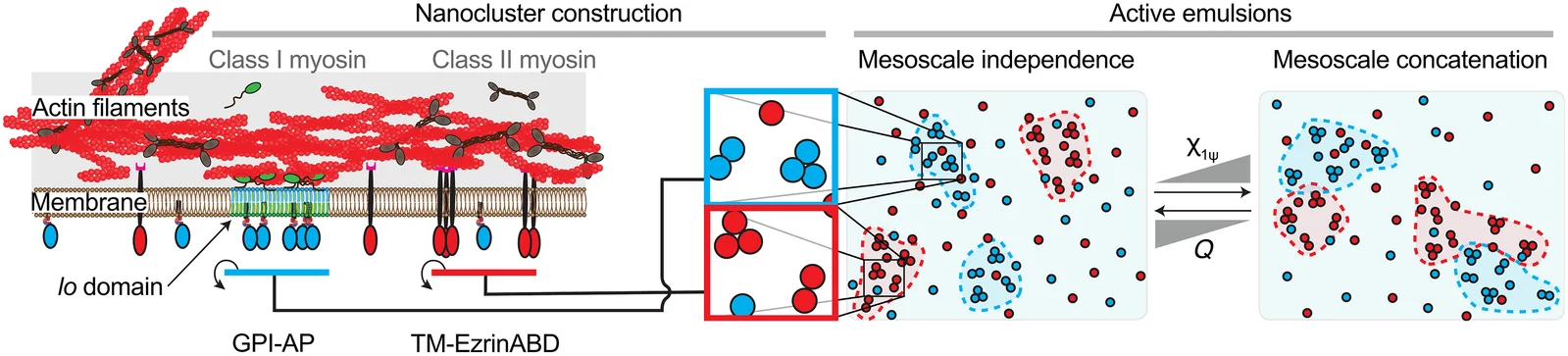
Model. Schematic showing the stratification of myosin motors at the PM driving the formation of nanoscale clusters of distinct components. These nanoclusters assemble into segregated mesoscale active emulsions that may be reversibly concatenated by increasing in-plane interactions strength $\left(\chi_{1\psi }\right)$ between the different membrane components even if they are coupled to different stress agents.

To test this prediction, we determined whether the distinct types of active emulsions could become concatenated (*45*) by increasing the mutual chemical affinities between the components of the two types of active emulsions [see the schematic in Fig. 7 (A and D)]. Palmitoylation is a common reversible posttranslational modification of TM proteins, which increases the affinity of the TM species for *lo* domains (*46*–*48*). Consistent with our prediction, we find an enhanced spatial overlap of strongly cluster-rich regions of palmitoylated CD44-GFP emulsions with the strongly cluster-rich regions of GPI-AP emulsions (TOS = 0.30 ± 0.19; Fig. 7, E and G); mutation of the palmitoylation sites in CD44 (CD44ΔPalm-GFP) led to a loss of this spatial correlation (TOS = 0.17 ± 0.25; Fig. 7, F and G). We note that while these data are consistent with our theoretical predictions, they do not uniquely validate this framework. Further experiments in particular reconstitutions of the two types of active emulsions in vitro and exploration of the outcome of the deliberate variation of the mutual chemical affinities as well as the parameter *Q* would be necessary to conclusively establish the theoretical framework being proposed. However, together, these data strongly suggest that the bifurcation of nonequilibrium membrane organization by active stresses arising from the two classes of nonmuscle myosins provides a chemically reversible mechanism to switch between segregated and concatenated states of mesoscale domains (see the model in Fig. 8).

### Functional implications of actively segregated nanoclusters

**Fig. 9.**
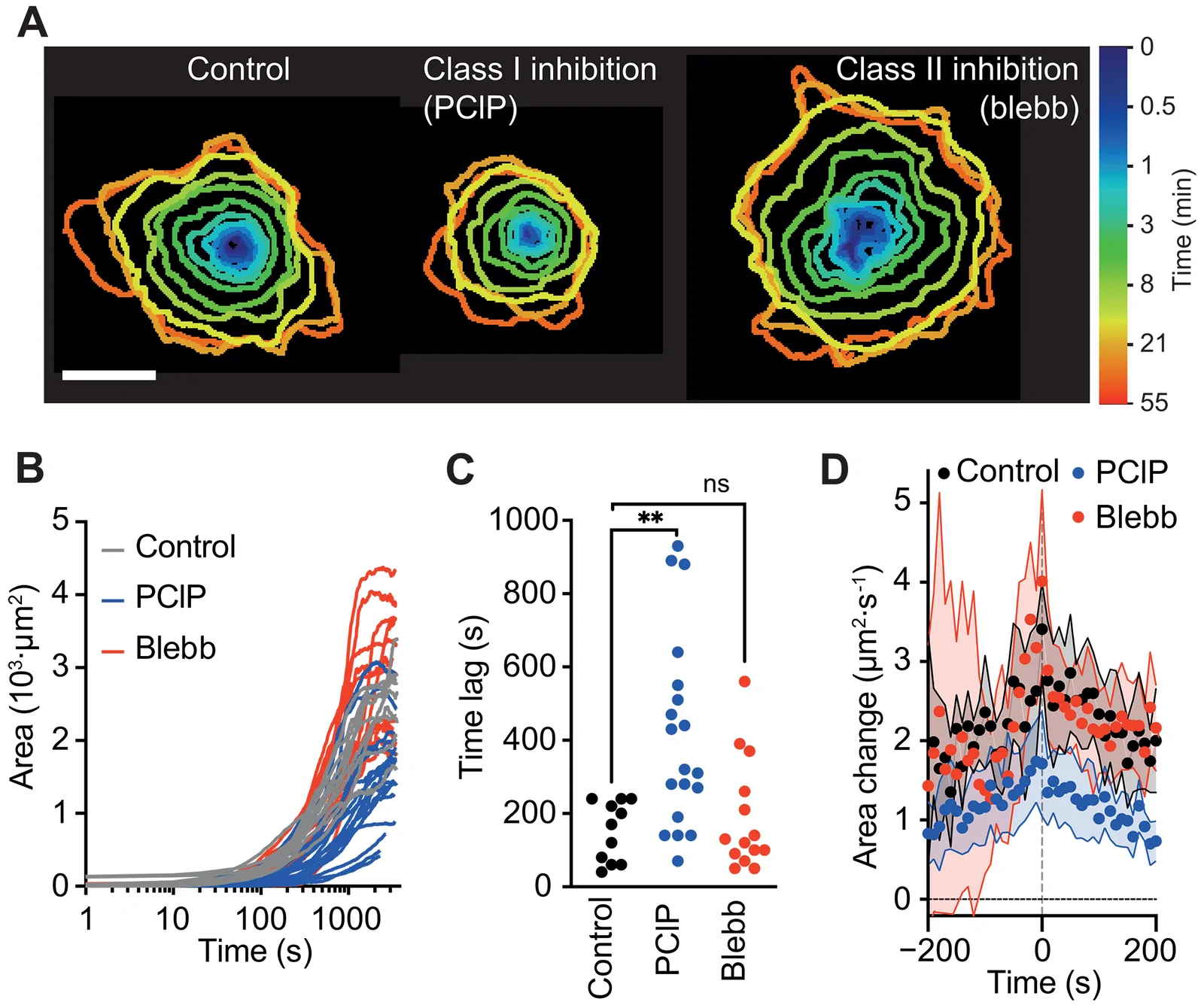
Functional consequences of myosin class I dependent membrane patterning on cell spreading. (**A**) Temporal evolution of spread area from cells treated with vehicle (DMSO; 1 hour), PClP (5 μM; 1 hour), or blebbistatin (blebb 50 μM; 1 hour) and adhering to fibronectin-coated surfaces imaged using interference reflection microscopy. (**B** to **D**) Graphs showing the time-dependent cell spread area per cell (B), time lag between cell attachment and cell spreading (C), and the extent of cell area increase [(D); means ± SD] during spreading of the same cells as in (B) where the maximum rate of area increase was shifted to *t* = 0. These data have been obtained from 11, 14, and 19 cells for DMSO, blebbistatin, and PClP, respectively; *n* = 3. Scale bar, 10 μm. ** and ns correspond to *P* < 10^−2^ and *P* > 0.05, respectively.

To address a functional relevance for the mechanistic bifurcation of the two myosin families in building membrane domains enriched in the distinct types of nanoclusters, we turned to cell spreading. During cell spreading, recruitment of class I myosins to the PM precedes activation of class II myosins (*49*, *50*), providing a temporal window to distinguish the functional contributions of the two myosin classes (see the schematic in fig. S7A). We have previously shown that following integrin activation, active emulsions enriched in GPI-AP nanoclusters are generated before cells spread on the substrate (*51*). Notably, this stage coincides with recruitment of class I myosin to the PM and precedes activation of myosin 2 (fig. S7A). Because GPI-AP active emulsions are required for cell spreading, and inhibition of class I myosins prevents their formation, we predict that this would consequently impair cell spreading. From the cell spreading area montages (Fig. 9A and fig. S7B), it is evident that inhibition of class I myosin abrogates the cell spreading response*.* The cell spreading response (Fig. 9B) displays both an increase in the lag time before cell spreading (Fig. 9C) and a decrease in the extent of area increase during spreading (Fig. 9D). This is consistent with class I myosin activity generating GPI-AP emulsions, which in turn are required for cells to spread upon activation of the integrin receptors (*51*). By contrast, inhibition of class II myosin does not increase the lag time nor abrogate cell spreading responses (Fig. 9, A to D); it augments the extent of spread area, likely by reducing cortical contractility (*52*). These results highlight the role of the two classes of nonmuscle myosins in functional segregation of specific components of the cell membrane.

## DISCUSSION

Nanoclusters such as those described here are emerging as major organizational units of larger-scale membrane platforms for signal transduction (*53*, *54*), cell-cell interactions (*31*, *55*), cell migration (*56*), and many other cellular processes (*6*, *57*). We provide evidence that multiple cell membrane constituents are patterned by distinct classes of ancestral myosin motors at the nano- and mesoscales. A stratified actomyosin cortex appears as a strategy for functional and tunable segregation of components in the membrane. Class II myosins embedded in the actin cortex transmit contractile stresses at a distance to membrane proteins attached to dynamic actin filaments in the cortical actin meshwork, generating the same nanoclusters and emulsions containing the actin-associated membrane proteins. On the other hand, class I myosins, when directly coupled to the membrane with their ability to recruit and move actin, generate lipidic nanoclusters, leading to functionally active emulsions at the mesoscale. Identifying such motorized PM-cytoskeleton linkers solves a long-standing question in the field of how outerleaflet lipids and proteins sense and register underlying actomyosin contractility to cluster at the PM. Given that class I myosin associates with PS at the inner leaflet and couples via transbilayer interactions with outerleaflet GPI-APs to create *lo-*ordered nanoscale domains (*16*, *19*), this is likelyto seed the generation of mesoscale *lo-*domains. This exemplifies the capacity of such an active process in building membrane regions of distinct chemical composition and properties.

This capacity emerges from a common physical mechanism connected to active stresses of polar filaments derived from their engagement with diverse myosin motors. The fact that several ATP-consuming machines (actin and at least two myosin classes) pattern membrane components into nanoscale clusters and consequently build active emulsions foregrounds the role of nonequilibrium mechanisms in structuring and organizing the PM. The mechanism described here (Figs. 7A and 8) also provides a tunable way to concatenate these domains via reversible posttranslational modifications (e.g., palmitoylation) that confer weak affinity interactions between components of the distinct emulsions (*45*).

Together, our findings establish class I myosins as active membrane-cytoskeleton linkers that drive the formation of lipid nanoclusters and active emulsions, yet several mechanistic questions remain unresolved. Although our data support interactions between class I myosins and multiple anionic lipids, the molecular basis of these interactions remains to be experimentally established, including their affinity, stoichiometry, and regulation within the PM. Likewise, the theoretical framework presented here has been tested under a specific set of experimentally accessible conditions. Although it provides a physical basis for understanding interactions between multicomponent active emulsions, a broader exploration of its predictions will be required to establish its generality. In particular, it will be important to examine how the framework extends across different membrane components, interaction strengths, activity-coupling strengths, motor activity regimes, and cytoskeletal architectures.

Despite these open questions, the patterning potential of the mechanism presented here is far-reaching; the association of class I myosins with specific lipids (i.e., PS and PIP_2_ for Myo1C) and membrane receptors [i.e., Myo1B to EphRB (*58*)] and the class II myosin-infused cortex with many proteins with actin-binding capacity are just some examples. A major contribution to this canvas is also the broad diversification potential of the proposed active mechanisms at the level of actin binding, positioning, and mechanics (*59*). We speculate that the segregation potential of this ATP-fueled fabric is made available for the crucial purpose of modulating information transduction, because it can be regulated in space and time and enable chemically reversible concatenation of diverse domains during the construction of signaling cascades (*57*).

## MATERIALS AND METHODS

### Materials

Tables listing reagents and their source, cell lines, plasmid DNAs, *Drosophila* strains, and simulation parameter are provided in tables S1 to S5.

### Methods

#### Cell culture, transfection, and labeling for live imaging

TM- or GPI-anchored fluorescent reporters were stably expressed in CHO cells (*14*). CHO-derived cell lines were maintained in Ham’s F12 media supplemented with 10% fetal bovine serum (FBS) and 1% penicillin-streptomycin-glutamine cocktail and with appropriate selection antibiotics. The maintenance media were free of phenol red, and where the FR domain was used for labeling, the maintenance media were additionally folate-free. The FBS used for these cells was dialyzed as described earlier (*36*). Human U2OS cell lines, U2OSGG cells stably expressing GFP-GPI (*51*), were maintained in McCoys, supplemented with 10% FBS and 1% penicillin-streptomycin-glutamine cocktail and with appropriate selection antibiotics.

Cells were plated in 35-mm glass-bottom dishes, 48 hours before live imaging, in maintenance media without selection antibiotic. Where applicable, transient transgene expressions were carried out by transfection of cells with Fugene6 following the manufacturer’s guidelines. When required, human fibronectin (hFN)–coated dishes were prepared by incubating the glass-bottom dish for 1 hour at 37°C with hFN (10 μg/ml) in phosphate-buffered saline.

Cells were imaged in M1 buffer (20 mM Hepes, 150 mM NaCl, 5 mM KCl, 1 mM CaCl_2_, and 1 mM MgCl_2_, adjusted to pH 7.4) supplemented with glucose (2 mg/ml; M1G). In the case of fluorescent protein–tagged membrane proteins, cells were incubated with cycloheximide (100 μg/ml) in culture media for at least 3 hours before imaging. For drug and inhibitor treatments, the cells were incubated at the desired concentration and indicated time before bringing the dish to the microscope for imaging. FRs were labeled by incubating the cells with 400 nM pteroyl-lysine-BODIPY-TMR (PLB) in M1G at 37°C for 10 min as the final step in the presence of the inhibitor or its absence.

#### Measuring relative localization of class I and II myosins

CHO cells that were plated for 2 days were cotransfected and grown for an additional 12 to 16 hours with plasmid DNAs encoding GFP-Myo1C and mCherry-Myo2A, de-adhered with TrypLE, pelleted down, and replated on hFN-coated dishes. Replating ensured that most cells were individually adhered and did not have cell-cell contact regions, which allowed optimal optical segmentation of single PM slices. After 2 hours, the cells were labeled with MemBrite Fix 405/430 following the manufacturer’s guidelines and subsequently fixed with 2% paraformaldehyde in M1G for 20 min at room temperature. After a permeabilization step with 0.1% Triton X-100 for 15 min, the cells were labeled with 200 nM Abberior STAR-635-phalloidin, washed three times, and imaged on a Zeiss LSM880 confocal microscope equipped with an Airyscan detector using a 63×, 1.4–numerical aperture (NA) oil objective. Because only two dyes can be detected during a single Airyscan *z*-stack, each cell was imaged using three spectral combinations (with Zeiss Airyscan deconvolution lateral and axial pixel sizes): GFP-UV (35.3 and 140 nm), GFP-Far Red (42.5 and 170 nm), and Far Red-mCherry (48.9 and 180 nm). Using 100-nm TetraSpeck beads, the three combinations were corrected for any spatial offsets. Next, between two and nine line profiles of around 6 μm in length were selected per cell going from extracellular to the cell interior passing the PM. For each line profile, the peak, corresponding to the position of the labeled component, was determined by fitting with a Gaussian function, and the lateral shift between the components was calculated. Starting from the membrane label (UV), the Myo1c position (GFP) was determined ($d_{\text{mem}\to \text{myo}1c}$); next, the phalloidin-labeled F-actin position (Far Red) was determined with respect to Myo1c ($d_{\text{myo}1c\to f-\text{actin}}$); and last, the Myo2a (mCherry) position with respect to F-actin was measured ($d_{f-\text{actin}\to \text{myo}2a}$). All the measured distances were calculated with respect to the membrane position (Fig. 1D). Each extracted line profile was shifted with respect to the membrane peak position at 1-μm intervals, all 69 line profiles were averaged, and their means ± SD are displayed in Fig. 1C.

#### Inhibitor treatments

For cholesterol depletion, cells were exposed to 10 mM methyl-β-cyclodextrin (MβCD) in M1G for 45 min with three buffer changes every 15 min at 37°C. Class II myosin was inhibited using either 100 μM blebbistatin or a cocktail that includes 20 μM ML7 and 20 μM Y-27632 in M1G for 1 hour at 37°C. Class I myosins were inhibited by incubating the cells for 1 hour with 5 μM PClP (*37*) at 37°C.

#### Steady-state emission anisotropy measurements

Emission anisotropy experiments were performed on a spinning-disk confocal microscope or on a total internal reflection fluorescence (TIRF) microscope, as detailed previously (*34*). Emission anisotropy images were collected by splitting the parallel ($I_{∥}$) and perpendicular ($I_{⊥}$) emission using a polarizing beam splitter and collecting the data on two cameras simultaneously.

The values at each pixel in the images that were acquired were all converted to photoelectrons. For data recorded with electron-multiplying charge-coupled device cameras, the experimental electron multiplying EM gain-factor was calculated directly from the image, as described previously (*60*). The pixel-wise gain for the scientific complementary metal-oxide semiconductor (sCMOS) was obtained using an experimentally precalibrated gain map (*61*). The raw image signal ($I_{∥}$ and $I_{⊥,\text{system biased}}$) was subsequently calculated by subtracting the experimentally determined dark image and dividing the image by the gain value (electron-multiplying charge-coupled device) or by the gain image (sCMOS). The raw images from the two polarizations were spatially aligned using iterative image registration combined with an affine transformation function (Matlab). Alignment corrections were kept at a minimal by experimentally aligning the images of subresolution beads before each imaging session. To correct for any system-dependent bias in the polarization detection resulting from the optics, a $G_{\text{factor}}$ image was determined using a 1 μM Rho6G solution where $G_{\text{factor}}=I_{∥,\text{Rho}6G}/I_{⊥,\text{Rho}6G}$, and the raw signal at the perpendicular channel ($I_{⊥,\text{system biased}}$) needs to be corrected for this bias: $I_{⊥}=G_{\text{factor}}\cdot I_{⊥,s\text{ystem biased}}$. After these corrections, the anisotropy was determined as follows

$$r=\frac{I_{∥}-I_{⊥}}{I_{∥}+2\cdot I_{⊥}}$$

To reduce the error in the anisotropy representation, both the $I_{∥}$ and $I_{⊥}$ were binned by summing up 3-by-3 neighboring pixels, improving the signal ninefold. The calculated anisotropy image was masked, showing only anisotropy values at pixels where there was a signal above the background.

The emission anisotropy values were calculated from the summed number of $I_{∥}$ and $I_{⊥}$ photons within square ROIs with 2.1-μm side lengths. ROIs were selected from intensity-uniform regions of the PM while avoiding areas within 2 μm of the cell edge. After background correction, the $I_{∥}$ and $I_{⊥}$ signals within each ROI were summed to calculate the anisotropy (r) and total intensity ($I_{T}=I_{∥}+2\cdot I_{⊥}$). For reliable measurements, ROIs were chosen to represent a single, in-focus PM sheet while minimizing contributions from membrane topology, intracellular membrane compartments, and imaging noise. Regions containing endosomal puncta or other prominent intensity features were excluded because these frequently exhibited elevated anisotropy values (fig. S8A). The ROI size (∼2 μm) was selected to be larger than the characteristic size of cluster-rich mesoscale domains (∼0.3 to 0.5 μm) while remaining sufficiently small to avoid sampling heterogeneous membrane environments. Because membrane organization is spatially heterogeneous, multiple ROIs were acquired from each cell to adequately sample the distribution of membrane organization. Approximately 10 ROIs per cell provided a robust estimate of the anisotropy-intensity relationship while minimizing oversampling of the most common mid-intensity range (fig. S8B). Bootstrapping analysis further showed that this sampling strategy yielded an error below 0.2 across the relevant intensity range (fig. S8C). Plots of anisotropy versus total fluorescence intensity (r versus $I_{T}$) were used to identify the intensity range suitable for quantitative comparisons, excluding low-intensity measurements that are dominated by photon noise and high-intensity measurements that are susceptible to concentration-dependent (trivial) FRET (*34*). Only ROIs within this range were used for comparisons between experimental conditions (Figs. 2, B and E; 3, B and E; and 5, B and C, and fig. S2, B to D and G). Absolute anisotropy values depend on experimental parameters including fluorophore properties, microscope NA, and excitation polarization. Consequently, anisotropy values were interpreted relative to internal controls acquired under identical imaging conditions rather than by a direct comparison of absolute anisotropy values across experiments.

#### Emission anisotropy upon photobleaching

A powerful method to determine whether the measured depolarization of emission anisotropy is a result of the occurrence of homo-FRET is to follow the anisotropy during photobleaching. During the photobleaching process, fluorophores undergo photobleaching in a stochastic manner; therefore, the number of unbleached fluorophores in the same nanocluster reduces (fig. S1A). For small cluster sizes as in the case of the nanoclusters under study, the measured anisotropy therefore increases linearly with the photobleaching extent (*34*); for the situation where there are only monomers, there is no change in emission anisotropy. Only fluorophores that cannot function as an energy acceptor after photobleaching can be used for these experiments; thus, this excludes extracellularly positioned GFP because, in nonreducing environments, the chromophore gets trapped in a dark state that can still participate in the energy migration process (*62*). Therefore, all our photobleaching experiments were performed with the PLB-labeled folate analogs [see example, sequences in fig. S1 (B and C)], where the fluorophores were photobleached using a 561-nm laser power at 20 to 30 mW, and 50 to 100 frames (100- to 200-ms integration time) were acquired at 0.5 to 2 Hz to measure emission anisotropy during this process. The intensity trace from each ROI, chosen from regions that were relatively uniform in intensity and had minimal amounts of bright spots, was background corrected, and the calculated anisotropy was plotted with respect to the photobleaching extent (fig. S1D), defined as 1−IT(t)I0, with $I_{T}\left(t\right)$ as the total intensity at frame *t* and $I_{0}$ being the total intensity at frame *t* = 0. A line was fitted to each ROI time series with “Photobleaching extent” < 0.6, and the slopes were recorded (fig. S1, D and E). Data on the slope per cell were combined from multiple cells and experiments and compared using the Mann-Whitney rank test (Figs. 2, C and F, and 3, C and F, and fig. S1E).

#### Drosophila melanogaster RNAi knockdown and GFP-GPI anisotropy

*Drosophila melanogaster* flies were maintained at 22°C in glass vials containing standard fly media. The fly line containing both collagen-GAL4 (gift from C. Dearolf) and UAS-GPI::EGFP (gift from S. Eaton) was generated in the lab. Class I and II myosin RNAi lines were obtained from VDRC and crossed with the generated transgenic GPI::EGFP line. Hemocytes were dissected from six third-instar larvae from each cross, as previously described (*31*). Briefly, third instar larvae were surface sterilized, and hemolymph was collected by puncturing the integument using dissection forceps into 150 ml of M1G [adjusted to pH 6.9 and additionally supplemented with bovine serum albumin (1 mg/ml)]. The medium with cells was resuspended, and the cells were allowed to spread for 1 to 2 hours on a glass-bottom dish. Emission anisotropy imaging of GPI::EGFP was performed on a TIRF microscope, and data were analyzed as described.

#### siRNA targeting of class I nonmuscle myosin

U2OSGG cells were set to deplete class I myosin proteins. For single isoform knockdown, U2OSGG cells were cultured in six-well plates and transfected using DharmaFect with siRNA pools against MYO1B (25 nM), MYO1C (25 nM), or MYO1D (25 nM) for 48 hours to achieve >75% knockdown of the each of the targeted isoforms. To knock down all three isoforms, 25 nM of each siRNA pool against MYO1B, MYO1C, and MYO1D was cotransfected. Cotransfections were performed thrice over two passages to achieve maximal knockdown of all three isoforms. Transfected cells at each time point were trypsinized and plated for imaging and/or harvested for protein analysis. Data in fig. S2 (D to H) correspond to anisotropy experiments and immunoblots from cells at the third passage. Nontargeting controls supplied along with the siRNAs were used for every experiment at similar concentrations.

#### Immunoblotting

U2OSGG cells were harvested after each transfection period. Protein lysates were prepared by homogenizing cells in radioimmunoprecipitation assay buffer with protease inhibitors. Protein content was estimated by a bicinchoninic acid estimation kit as per the manufacturer’s protocol. A total of 15 to 20 μg of protein was loaded onto 10% tris-glycine polyacrylamide gels. After transfer, polyvinylidene difluoride membranes were blocked with Everyblot blocking buffer followed by overnight incubation with primary antibodies, washing, and secondary antibody incubation for 1 hour at room temperature. Membranes were probed with Clarity Max ECL reagent. The expression of class I myosin proteins was normalized to tubulin loading controls in the same lanes. Normalization with another housekeeping control, GAPDH (glyceraldehyde-3-phosphate dehydrogenase), yielded similar expression levels.

#### Bleb cross-linking and analysis

CHO cells [TrVb line, stably expressing FR (FR-GPI) or a TM actin-binding variant (FRTM-Ez-AFBD*] were transfected with GFP-Myo1c, GFP-Myo1b, or LactC2-GFP for 12 to 15 hours. Blebs were then generated on these cells by incubating with 14 μM jasplakinolide at 37°C for 30 min in M1G. The cells were then incubated with a MOV18 primary antibody against the FR at 5 μg/ml for 1 hour on ice. This was followed by incubation with a secondary antibody (2 μg/ml) for 30 min on ice. Samples were imaged immediately after the washing steps to avoid washing away the weakly cell-attached blebs. Images were obtained on a Zeiss LSM 780 confocal microscope at room temperature using a 63×, 1.4-NA oil objective by sequential imaging of the cross-linked antibody in far-red (647-nm excitation) and the transfected protein in green (488-nm excitation). The blebs were analyzed using a custom Matlab routine that fits the diffraction-limited bleb contour, followed by obtaining the average intensity profile along the 5-pixel broadened bleb circumference of both channels. The signal from both channels was cross-correlated, giving a single Pearson’s coefficient for each bleb (Fig. 4F).

#### Multiwavelength emission anisotropy correlation: Nanoscale and mesoscale

To correlate emission anisotropy from two distinct membrane components, sequential image acquisition was conducted by changing the excitation wavelength and the emission filter. Images were collected as fast as the microscopy system could switch excitation and emission filters (∼1.0 s). For the nanoscale analysis, relating anisotropy of one component to the relative local abundance of a second membrane component, square ROIs with vertices of 2.1 μm in length were selected as indicated above. Only those ROIs where $I_{T}$ versus anisotropy was free from noise and trivial FRET were taken further for analysis (*34*). Next, the total intensity of the other channel was determined and used to set a cutoff intensity for the second channel while reporting on the anisotropy of the first channel [see Fig. 5 (B and C) and fig. S4B]. Alternatively, it was used to calculate the local intensity ratio, $\frac{I_{T,\text{ch}2}}{I_{T,\text{ch}1}}$ and plotted against the anisotropy calculated from the first channel $r_{\text{ch}1}$ (Fig. 6A).

To calculate the covariance of anisotropy values between innerleaflet myosin 1 and outerleaflet GPI-AP, 3-by-3 pixels of the raw images were binned to increase the photon count from which the anisotropy was calculated. We next selected a single drawn ROI per cell containing the largest area with relative uniform membrane fluorescence and cross-correlated the anisotropy values of the two channels, giving a single Pearson’s coefficient for each cell (Fig. 4C).

For mesoscale domain co-occurrence analysis, anisotropy images from the two fluorescence channels were analyzed using the TOS method described previously (*44*), with minor modifications to accommodate anisotropy-based pixel selection. Anisotropy was first calculated for every pixel in each fluorescence channel to generate two anisotropy maps. For each ROI, the mean anisotropy (〈r〉) and SD [σ(r)] of the pixel distribution were determined and used to normalize the anisotropy values of individual pixels: $\frac{r_{\text{pixel}}-\langle r\rangle }{\sigma \left(r\right)}$. This normalization enables a direct comparison of anisotropy deviations between channels and across cells (Fig. 6B and fig. S5, A and B). Pixels were then classified according to their normalized anisotropy using threshold values defined as multiples (n) of the SD from the mean. Low-anisotropy pixels [r<〈r〉−n·σ(r)] correspond to progressively cluster-rich regions, whereas high-anisotropy pixels [r>〈r〉+n·σ(r)] represented progressively cluster-poor regions. The threshold parameter n was varied between 0 and 3, producing graded categories ranging from weakly to strongly cluster-rich or cluster-poor regions [see colored arrows in fig. S5 (B and C)]. A TOS matrix was then generated by computing the TOS for all combinations of threshold settings between the two channels (fig. S5, D to F). TOS quantifies the weighted area overlap between threshold-selected pixels after correction for the overlap expected from a random spatial distribution. Values range from −1 (less overlap than expected by chance), through 0 (random overlap), to +1 (complete overlap). Unless otherwise indicated, comparisons were performed using the cluster-rich threshold category [dashed region in fig. S5 (E and F)], and the mean TOS value was used for statistical analysis. The fraction of pixels selected depended on the threshold applied [see example images in Figs. 6 (C to E) and 7 (E and F) and fig. S5C (top panels)]. Threshold combinations that yielded no selected pixels in either channel were excluded from averaging. Further details about quality control and randomized distribution of pixel anisotropy values are described in the Supplementary Text.

#### Cell spreading experiment and analysis

Fibronectin-coated glass dishes were prepared as mentioned earlier. The cells were deadhered with TrypLE, pelleted down, resuspended in incomplete media, and left for 30 min at 37°C for recovery. Next, the cells were treated by supplementing the suspensions with the appropriate amount to obtain a final concentration of 0.1% dimethyl sulfoxide (DMSO), 50 μM blebbistatin, or 5 μM PClP and left at 37°C for 60 min. Just before the experiment, the hFN-coated dishes containing the appropriate solution were placed in a preheated microscopy chamber at 37°C (OKO Labs). Images at a 0.1-Hz frame rate were recorded using interference reflective microscopy upon adding the pretreated cells to the dish. Each identified cell in the image series was analyzed separately using an available MatLab-based software that determined the cell contour at each frame (*63*). The resultant area-versus-time curves allowed for the calculation of the spreading lag time, spreading rate, and final spread area.

#### Interference reflection microscopy

A 10/90 (reflection/transmission mirror, Thorlabs) unpolarized light filtered at 480/20 (Chroma) from a light-emitting diode source (pE-400, CoolLED) was directed to the sample using a 60×, 1.49-NA objective. Reflected light was collected through the same objective and imaged with an sCMOS camera. Through destructive interference of the reflected light, adhering cells were detected as regions of reduced intensity on the camera.

#### Statistics and data reproducibility

A Mann-Whitey ranked test was performed for comparing distributions of anisotropy values, Pearson’s correlation coefficient values, and TOS values. The data associated with each figure have been deposited in a Zenodo repository (DOI: 10.5281/zenodo.21217537) as well as the analysis files together with a representative dataset (DOI: 10.5281/zenodo.21241290).

## Supplementary Materials

### This PDF file includes:

Supplementary Text Figs. S1 to S8 Tables S1 to S5 References
