# Supplementary material for: Diverse ancestral myosin motors generate and segregate distinct types of nanocluster-rich domains at the plasma membrane
Source: Sci Adv. 2026 Sep 11;12(37):eaeb8889. doi: 10.1126/sciadv.aeb8889 (PMC13564907; doi:10.1126/sciadv.aeb8889)
Supplement: Supplementary file 1 [file sciadv.aeb8889_sm.pdf]

Science Advances
AAAS

# Supplementary Materials for

## Diverse ancestral myosin motors generate and segregate distinct types of nanocluster-rich domains at the plasma membrane

Parijat Sil *et al.*

Corresponding author: Satyajit Mayor, mayor@ncbs.res.in, satyajit.mayor@warwick.ac.uk

*Sci. Adv.* **12**, eaeb8889 (2026)
DOI: 10.1126/sciadv.aeb8889

**This PDF file includes:**

Supplementary Text
Figs. S1 to S8
Tables S1 to S5
References

**Supplementary Text**

Active Flory-Huggins theory for a three-component membrane coupled to the actomyosin cortex:

In previous studies(*26*, *64*), we developed an Active Flory-Huggins (AFH) theory for a multicomponent membrane interacting with the actomyosin cortex comprising a single myosin species. Here we extend our framework to include two myosin species, namely the class I and class II myosin, with differential turnover rates and contractility.

Consider a planar membrane, composed of a ternary mixture of saturated lipids (*lo* component, that includes GPI-APs), unsaturated lipids (*ld* component) and transmembrane proteins (TM-EzAFBD), with area fraction $\phi_1$, $\phi_2$ and $\psi$ respectively, such that $\phi_1 + \phi_2 + \psi = 1$. At temperature above $T_c$, the membrane is in a homogeneous, mixed phase. At temperatures below $T_c$, the mixture undergoes an *lo-ld* equilibrium phase separation, with the saturated lipids predominantly in the liquid-ordered (*lo*) phase and the unsaturated lipids in the liquid-disordered (*ld*) phase.

On coupling to the actomyosin cortex, the *lo* component experiences a force from active stresses generated through class I non-muscle myosin and the direct actin binder TM-EzAFBD experiences a force from the active stresses generated through class II non-muscle myosin. The dynamics of the area fractions of the components follow from local force balance and mass conservation (*26*, *64–66*),

$$\frac{\partial \phi_1}{\partial t} = \nabla \cdot \left[-L\left(\nabla \mu_\psi - K_{II}\, \nabla \cdot \sigma_{II}\right) + M_1(\nabla \mu_1 - K_I\, \nabla \cdot \sigma_I)\right]$$

$$\frac{\partial \psi}{\partial t} = \nabla \cdot \left[-L\left(\nabla \mu_1 - K_I\, \nabla \cdot \sigma_I\right) + M_\psi\left(\nabla \mu_\psi - K_{II}\, \nabla \cdot \sigma_{II}\right)\right] \qquad (1)$$

where the exchange mobilities(*65*, *66*) are given by $L \propto \phi_1\, \psi$, $M_1 \propto \phi_1(1-\phi_1)$, and $M_\psi \propto \psi(1-\psi)$, and the currents in the right-hand side of the above equations have contributions from the equilibrium exchange chemical potentials $\mu_1$ and $\mu_\psi$, and the nonequilibrium active contractile stresses $\sigma_I$ and $\sigma_{II}$, arising from the action of class I and class II myosin, respectively. The exchange chemical potential is obtained from the Flory-Huggins free energy $F$,(*64*) specifically,

$$\mu_1 = \chi_{12}(\phi_2 - \phi_1) + \left(\chi_{1\psi} - \chi_{2\psi}\right)\psi + \ln(\phi_1/\phi_2) - \kappa\nabla^2(\phi_1 - \phi_2)$$

$$\mu_\psi = \chi_{2\psi}(\phi_2 - \psi) + \left(\chi_{1\psi} - \chi_{12}\right)\phi_1 + \ln(\psi/\phi_2) - \kappa\nabla^2(\psi - \phi_2) \qquad (2)$$

where $\chi_{12}$, $\chi_{1\psi}$, $\chi_{2\psi}$ are the Flory-Huggins interaction parameters between the membrane components controlling affinity of *lo* with *ld*, *lo* with TM-EzAFBD, and *ld* with TM-EzAFBD, respectively.

The parameters $K_I$ and $K_{II}$ characterise the coupling between class I myosin with *lo* component and class II myosin with TM-EzAFBD, respectively. The active cortical stresses $\sigma_{I,II}$ in equation (1) are evaluated from the active hydrodynamic equations describing the actomyosin cortex (*64*) in terms of the local concentration of actomyosin (equations for mass balance) and the cortical velocities of bound class I and class II myosins (equations for force balance). The form of the

active stresses, exerted by bound class I and class II myosins (Supplementary Fig S6A, D) is given by:

$$\sigma_I = \left(\frac{\zeta_I\, c_I}{1 + c_I} - Q_I c_I c_{II}\right) I$$

$$\sigma_{II} = \left(\frac{\zeta_{II}\, c_{II}}{1 + c_{II}} - Q_{II} c_I c_{II}\right) I \qquad (3)$$

where in the absence of any long range orientational order, the active stresses are isotropic (thus in the above equation, $I$ is the identity matrix). The first term shows the usual saturating form with $\zeta_I, \zeta_{II} > 0$ for contractile stresses (*67*). The second term represents a potential inhibition of the local activity of bound myosin motor I due to the presence of myosin motor II (and *vice versa*). While in general $Q_I$, $Q_{II}$ can have different values, we have, for convenience, taken them to be the same and equal to $Q$.

The mass balance equations express the balance between advection and turnover of the bound myosin species. The turnover of myosin has contributions from unbinding and binding. Here, we take the unbinding rate to be constant and choose the following simple form for the binding rates,

$$k_{bI}(\phi_1, \psi, c_I, c_{II}) = A_I \phi_1 - B_I c_{II}$$

$$k_{bII}(\phi_1, \psi, c_I, c_{II}) = A_{II} \psi - B_{II} c_I \qquad (4)$$

which includes specific binding $A_I, A_{II}$ to the membrane components, *lo* and TM-EzAFBD, respectively, thereby generating positive feedback, and competitive binding $B_I, B_{II}$ between the myosin species.

This completes our description of the active Flory-Huggins (AFH) theory, for the three-component membrane coupled to an active cortex comprising two myosin species. The explicit form of the active hydrodynamic equations of the actomyosin cortex are presented in (*64*). Using this, we study the steady state configurations of the membrane composition at a fixed temperature below and above $T_c$ starting from mixed initial condition. As seen in (*26*, *64*) even at temperatures above $T_c$, we observe mesoscale segregation of the *lo* and *ld* components, arising from active stresses and lateral interactions between the lipid species. Even though TM-EzAFBD has no affinity to either *lo* or *ld* components, it segregates from the *lo* components (Fig S6G and Supplementary Fig S6B,C) due to differential active stresses generated by myosin I and myosin II. The extent of overlap between the *lo* and TM-EzAFBD domains depends on the Flory interaction parameters $\chi_{1\psi}$, $\chi_{2\psi}$ (Fig 7G,H and Supplementary Fig S6E,F) and the actomyosin parameters $B_I, B_{II}$, and $Q$ (Fig 7H). As follows clearly from our discussion here and in the main text, the effect of changing these parameters on the segregation of the *lo* component and TM-EzAFBD domains is displayed in Supplementary Table 5 below. A striking observation is that the domains of *lo* and TM-EzAFBD exhibit strong density inhomogeneities which we refer to as *granularity* (see main text).

Threshold Overlap Score (TOS) of anisotropy images

With TOS analysis(*44*) one can enquire whether a region in an fluorescence emission anisotropy image contains pixels of user-defined anisotropy values that preferentially overlaps with a coresponding region in a second fluorophore's emission anisotropy image that exhibits pixels of a certain anisotropy value, beyond that expected from random chance. TOS also eliminates much of the random noise in the data, coming from anisotropy values that are not of interest, by setting a threshold. Instead of choosing a single threshold we scan through the anisotropy distribution of both channels by setting multiple thresholds and for quantification we average over a range of thresholds. The two channels that were used were PLB-labeled FRTM-Ez-AFBD and GFP-GPI (Fig 6B-F, Supplementary Fig S5A-E) or PLB-labeled FR-GPI and GFP-CD44 (Fig 7E-G, Supplementary Fig S5F). Note that in the examples shown in Supplementary Fig S5C sup-panel (i) and (ii) display some overlapping pixels whereas there is an almost lack of overlapping pixels in subpanel (iii). These overlaps are contrasted to chance overlap(*44*).

For quality control, a TOS matrix was first generated between two images that were acquired sequentially. Here, the first channel is an anisotropy image at t=0 and the second channel is an anisotropy image that is shifted in time by 2 seconds assuming minimal re-organisation of myosin activity during this time frame. The scores obtained between these two images will be the maximally obtainable TOS (Supplementary Fig. S5D,E). Cells with no positive TOS between the anisotropy-low regions (or anisotropy-high regions) of sequential images of either of the channels were not taken forward for analysis. Day-to-day variability was non-significant for any of the four quadrants of the TOS matrix (Supplementary Fig. S5G).

Simulations of randomly placed pixels chosen from a gaussian distribution of anisotropy values resulted in a TOS matrix that was approximately 0 for all thresholds (Supplementary Fig. S5H). Randomization of the pixels from the experimentally obtained data also resulted in TOS values close to 0, however, the regions with a low number of pixels (the four edges; the most stringent thresholds) contained the largest standard deviation in TOS-values (Supplementary Fig. S5I) and typically had an underestimated TOS-value. Quantification of TOS was therefore obtained from the average values of the bottom left quadrant of the TOS matrix (overlap between low-anisotropy regions) excluding the most stringent four bins (dashed area in the TOS matrix of Supplementary Fig S5E, F).

Sequence comparison and *in silico* analysis of class I myosin interaction with anionic lipid species.

Sequence comparison, identification of conserved residues in lipid binding and actin-binding domains of myosin 1C homologs and percentage sequence homology between the lipid-binding and actin-binding domains of myosin 1c homologs from different organisms (mouse, human and drosophila and dictyostelium) were done with Uniprot alignment tool using ClustalΩ (*68*) via the MPI Bioinformatics Toolkit**.** Ribbon diagrams and the location of the surface charge maps in the 3D structure of Myo1B and C were generated with pymol using the tails domains of Myo1B (Uniprot ID Q05096, Isoform B) and Myo1C (Uniprot ID Q9WTI7, Isoform 2).

To probe potential protein-lipid interaction sites, we used the CCD2MD lipid library(*43*) together with AlphaFold3(*42*) to co-fold the above tail domains of Myo1B and Myo1C in the presence of anionic lipid molecules (PIP2 and DOPS in a 1:10 and 2:10 stoychioimetry). Across all generated models, PIP2 consistently localizes to a specific binding pocket within the PH domain. In Myo1B PIP2 binds to a conserved cluster of basic residues (Lys995, Asn997, Arg998, Arg1006, Lys1026, Lys1060), while in Myo1C, it engages a corresponding cluster of residues (Lys927, Asp929,

Arg930, Arg936, Lys989), indicating the presence of a specific and preferred phosphoinositide-binding site.

In contrast, PS lipids do not occupy a single well-defined binding pocket. Instead, PS molecules are distributed across multiple sites at the interface between the membrane and the tail domain. Some of these sites partially overlap with the PIP2-binding region. For example, in the models generated with a single PIP2 molecule, residue Lys931 in Myo1C is frequently engaged by PS, whereas in models containing two PIP2 molecules, this same residue preferentially coordinates the second PIP2. Other PS interactions extend toward adjacent regions, including areas near the IQ3 domain, consistent with a more diffuse and less specific binding mode.

This difference is supported by the predicted aligned error (PAE) analysis. The position of PIP2, as well as a subset of PS lipids located near the main binding pocket, shows low PAE (dark green), indicating high confidence in their relative positioning with respect to the protein (Supplementary Fig. S3D).

Consistent with these observations, electrostatic surface representations of the PH domains in both Myo1B and Myo1C reveal a prominent positively charged region corresponding to the specific PIP2-binding pocket, alongside additional dispersed basic patches that could support electrostatic interactions with anionic lipids such as PS (Supplementary Fig. S3E).

Taken together, these results support a model in which Myo1B and Myo1C contain a single, specific binding site for PIP2, while PS interacts more diffusely through multiple lower-specificity electrostatic contacts.

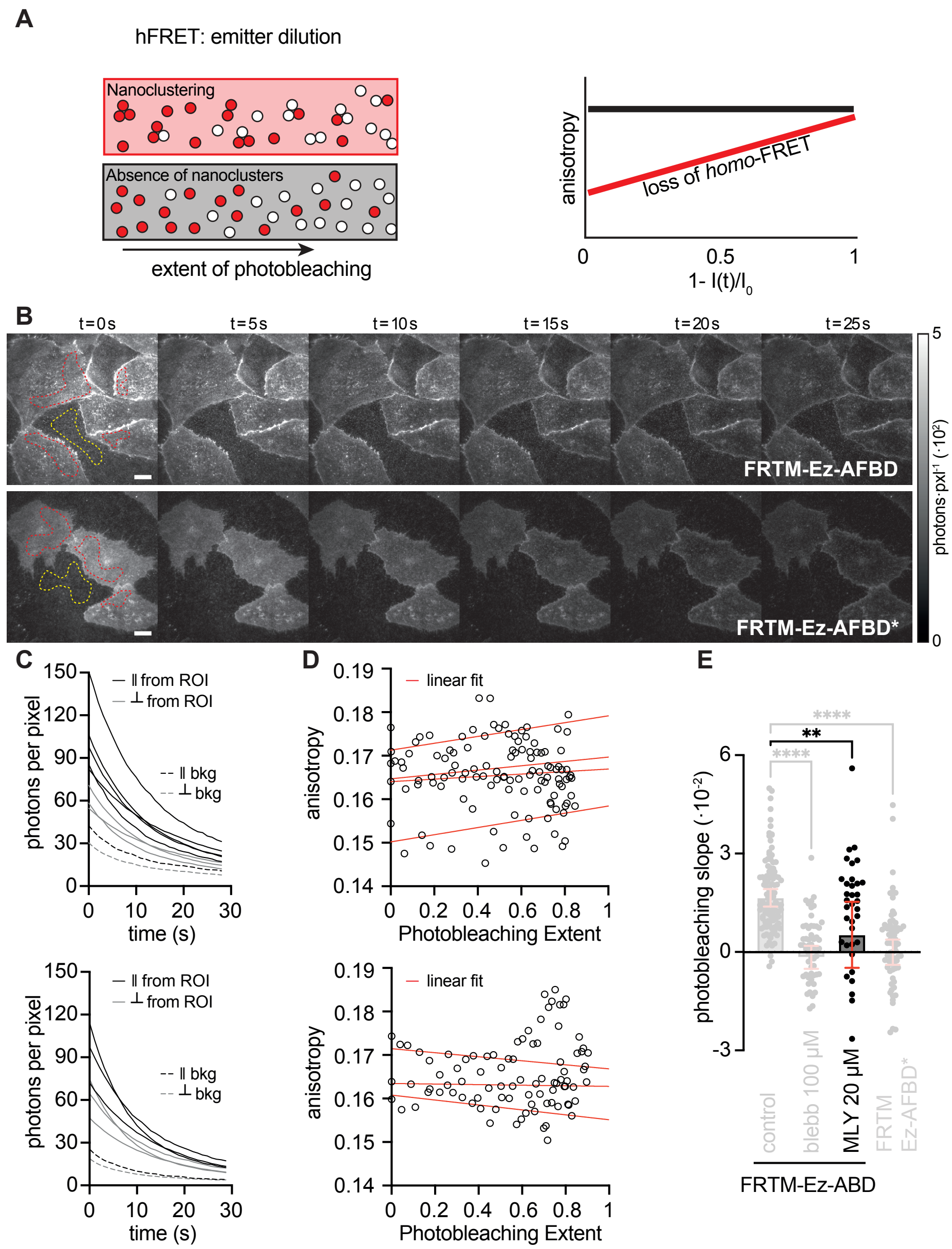


**Supplementary Figure S1. Photo-dilution experiments on FR-labeled cells and workflow to extract the anisotropy-photodilution slope. A)** Cartoons (left) provide a pictorial representation of molecules (red circles) organized as monomers with nanoclusters (pink background) or

monomers only (grey background) during photobleaching, which results in the dilution of fluorophores. Representative plot (right) of the change in fluorescence emission anisotropy of the monomers with nanoclusters (red) and monomer only (black) panels following photobleaching. At the start of the photobleaching process, the collection of monomers and nanoclusters (pink panel) have a lower collective anisotropy due to homo-FRET between fluorophores in nanoclusters compared to those that are present only as monomers (grey panel). Following photobleaching the progressive loss of fluorophores (indicated as open circles) results in only monomers remaining in both cases, and the emission anisotropy values equalize. The slopes of these plots provide a method to compare the extent of nanoclustering. **B)** Montages of total fluorescence intensity images (calculated through ⊥ and ∥ channels) from PLB-labelled FRTM-Ez-AFBD and FRTM-Ez-AFBD* show the loss of fluorescence as a function of progressive photobleaching. Regions of interest from cells are indicated by a red-dashed lines and the background region is selected with the yellow-dashed line. **C)** The traces from PLB-labelled FRTM-Ez-AFBD (top) and FRTM-Ez-AFBD* (bottom) cells capture emission intensity decays of the orthogonal polarisations from specific regions of interest indicated in panel B. **D)** Plots depict the change in emission anisotropy (y-axis) as a function of the extent of photobleaching (x-axis). Each region of interest is plotted as a scatter plot and fitted to linear equations (red lines; $y = mx + c$). **E)** Bar graph shows the mean (±s.d.) of the slopes extracted from the linear fits of the data derived from the FRTM-Ez-AFBD construct treated with MLY (38 cells, n=2). The grey bars depict data already shown in Fig. 2C to allow direct comparison. Scale bars, 10 μm. ****, and ** correspond to *p*-values of $<10^{-4}$, and $<10^{-2}$, respectively.

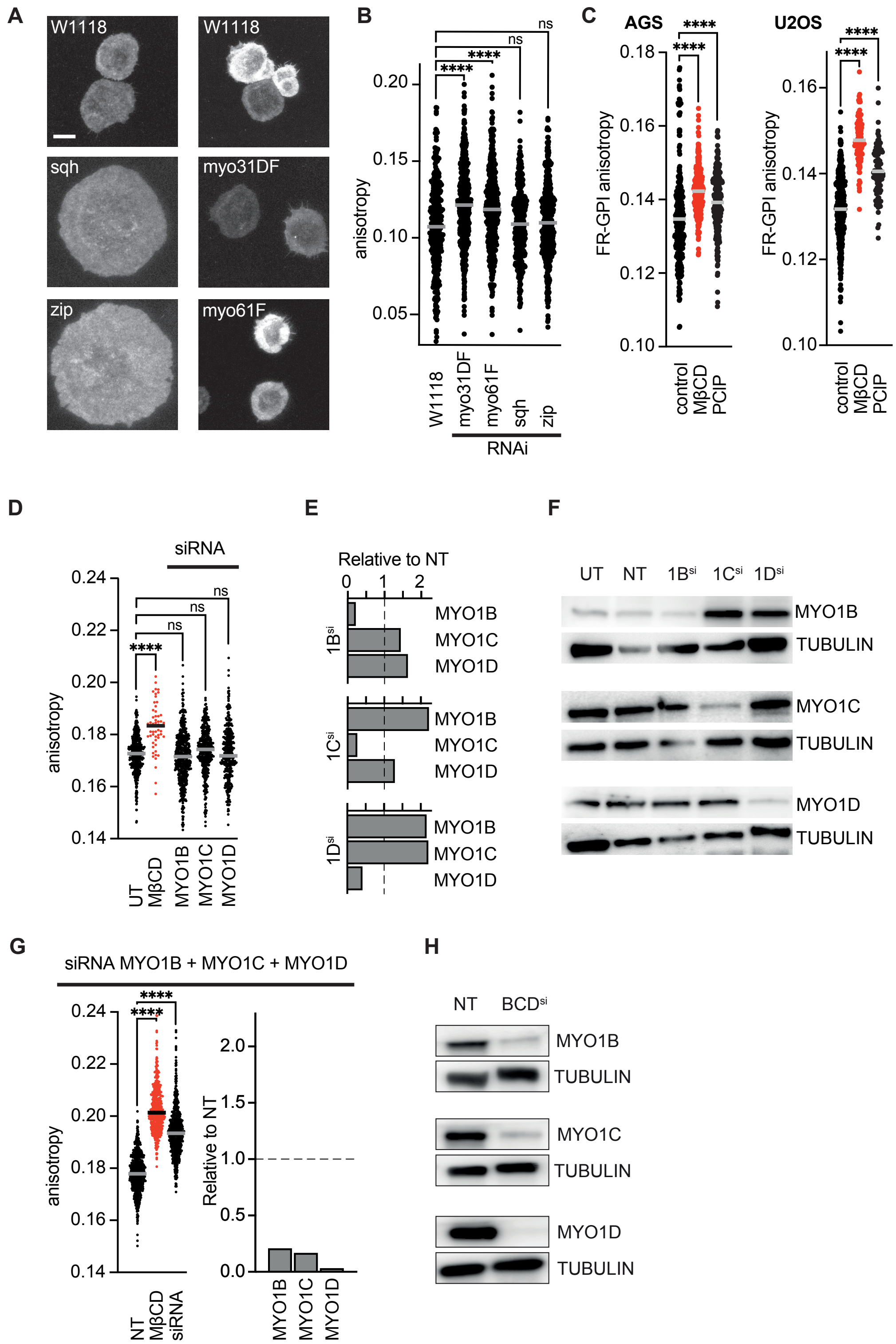

A
W1118
W1118
sqh
myo31DF
zip
myo61F
B
anisotropy
W1118
myo31DF
myo61F
sqh
zip
RNAi
ns
C
AGS
U2OS
CHO
FR-GPI anisotropy
mRubi-GPI anisotropy
control
MβCD
PCIP
D
siRNA
anisotropy
UT
MβCD
MYO1B
MYO1C
MYO1D
E
Relative to NT
1B^si
1C^si
1D^si
MYO1B
MYO1C
MYO1D
F
UT
NT
1B^si
1C^si
1D^si
MYO1B
TUBULIN
MYO1C
MYO1D
G
siRNA MYO1B + MYO1C + MYO1D
anisotropy
NT
MβCD
siRNA
Relative to NT
H
NT
BCD^si
MYO1B
TUBULIN
MYO1C
MYO1D

**Supplementary Figure S2. Myosin class I siRNA knock down. A)** Montages of total fluorescence intensity images (calculated through $\boldsymbol{I}_{\parallel}$ and $\boldsymbol{I}_{\perp}$ channels) of hemocytes derived from a *D. melanogaster* GPI::GFP transgenic crossing with a transgenic containing the indicated UAS-dsRNAi. **B)** Graphs showing anisotropy values from the corresponding GFP-GPI expressing hemocytes expressing the indicated dsRNAi. The data set was obtained from 55 cells (340 ROIs), 83 cells (477 ROIs), 77 cells (467 ROIs), 33 cells (348 ROIs), and 34 cells (452 ROIs) for the cross with W1118, myo31DF, myo61F, sqh, and zip, respectively. **C)** Graphs showing anisotropy values from cells treated with the vehicle (DMSO), MβCD (10 mM), or myosin 1 inhibitor (PClP at 5 μM). The data is collected from FR-GPI, labeled with PLB, and expressed in AGS cells (left graph: 21 cells {307 ROIs}, 28 cells {333 ROIs}, and 23 cells {280 ROIs} for control, MCD and PClP, respectively), FR-GPI expressing U2OS cells (middle graph: 23 cells {414 ROIs}, 9 cells {116 ROIs}, and 26 cells {91 ROIs} for control, MCD and PClP, respectively) and mRubi-GPI expressing CHO cells (right graph: 17 cells {177 ROIs}, 16 cells {143 ROIs}, and 20 cells {129 ROIs} for control, MCD and PClP, respectively). **D)** Anisotropy distributions of GFP-GPI in U2OS cells depleted of Myosin1 isoforms. Graph shows anisotropy values of cells that were treated with siRNA against Myo1B, Myo1C, or Myo1D. Anisotropy values of the siRNA treatments and cells treated with 10 mM MβCD to abrogate GFP-GPI clustering were compared to the untreated control (UT). The anisotropy data was compiled from 47 cells (468 ROIs), 27 cells (56 ROIs), 44 cells (484 ROIs), 29 cells (383 ROIs), and 29 cells (351 ROIs) for the untreated cells, MβCD, MYO1B siRNA, MYO1C siRNA, and MYO1D siRNA, respectively. **E)** Bar graph shows levels of MYO1B, C&D protein expression in U2OS cells that were treated with siRNAi against Myo1B, Myo1C, or Myo1D. Protein levels for Myo1B, Myo1C and Myo1D were normalized to the non-target control. **F)** Representative western blot showing the reduction of the respective myosin protein bands with the loading controls for the untreated cells, the non-target siRNA and the three individual myosin 1 siRNA. **G)** Graph shows anisotropy values of cells that were treated with a combined cocktail of siRNA against Myo1B/Myo1C/Myo1D compared to the non-target control and the 10 mM MβCD. The data was compiled from 40 cells (666 ROIs), 29 cells (456 ROIs), and 64 cells (711 ROIs) for the non-target siRNA, MβCD, and triple siRNA knock downs, respectively. Protein levels for Myo1B, Myo1C and Myo1D were also compared to the non-target control. Note that only when a triple siRNA cocktail is used, there is a significant increase in emission anisotropy comparable to that seen with the loss of clustering due to MβCD-treatment. **H)** Representative western blot showing the reduction of the respective myosin protein bands with the indicated loading controls. Scale bar, 10 μm. ****, and ns correspond to *p*-values of $<10^{-4}$, and $>0.05$, respectively.

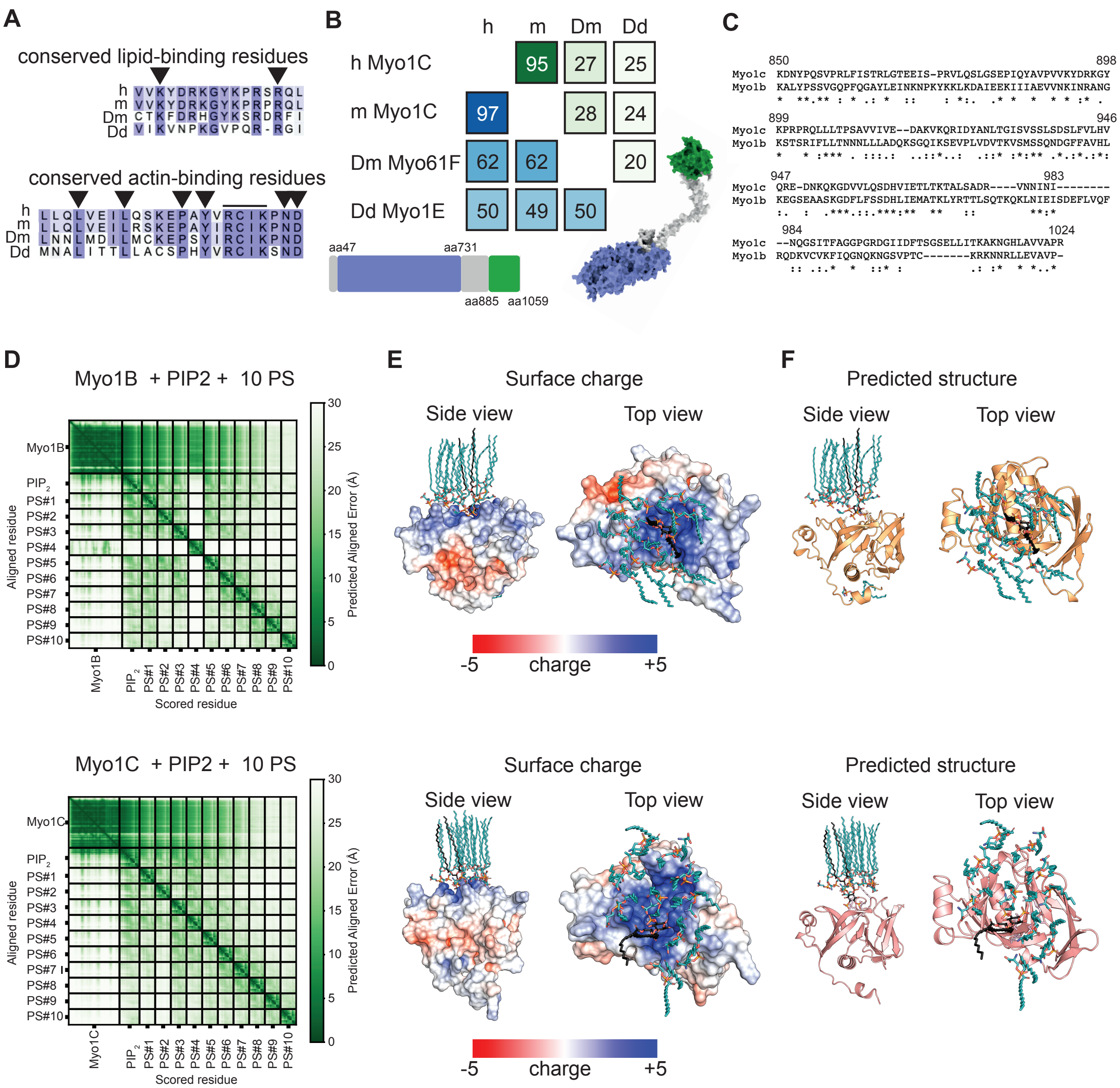

**Supplementary Figure S3. Myosin class I comparison. A)** Conserved residues of the lipid binding and actin binding domains (blue shades) of Myosin-1c homologs across, mammals (human, mouse, Chinese hamsters (CHO)), fruitflies (Drosophila) and protists (Dictyostelium) are highlighted in blue. **B)** Percentage sequence homology between Myo1c proteins across these species in the motor domain and tail region are depicted in various shades of green and blue, respectively. **C)** Amino acid alignment between the tail-domains of Myo1B and Myo1C showing overall conservation of amino acid and structure. **D)** Predicted aligned error (PAE) plots of Myo1B (top) and Myo1C (bottom) with the anionic lipids: 1 PIP2 lipid and 10 PS lipids. Low PAE values (dark green) indicate high confidence in their relative positioning with respect to the protein. **E)** Surface electrostatic potential of tail domains from Myo1B (top) and Myo1C (bottom). The tail domain is oriented to display the membrane interacting surface as a side view and face-on together with the anionic lipids: PIP2 in black and PS in cyan. **F)** Ribbon representation of the same as D.

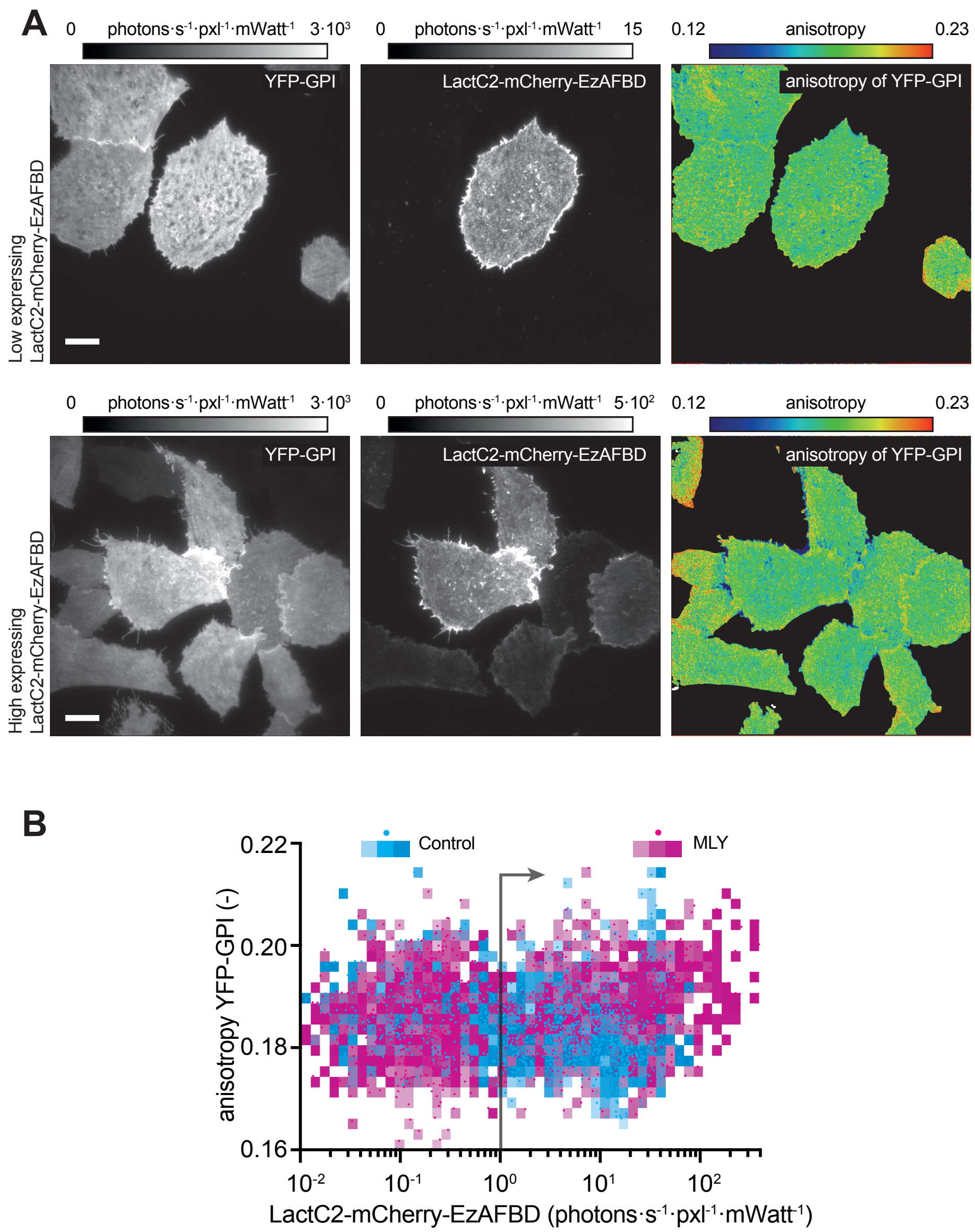


**Supplementary Figure S4. Co-expression of a synthetic PS-actin linker protein and GPI-AP. A**) Intensity and anisotropy (right panel) images of cells co-expressing YFP-GPI (left panels) and different levels of the PS-actin linker, mCherry-LactC2-EzAFBD (center panel). Intensity images display the total photon counts from each fluorophore. **B**) Scatter plot shows anisotropy of YFP-GPI as a function of the (local) expression level of the mCherry-LactC2-EzAFBD chimeric protein. In green are the ROIs from control cells and in red the ROIs from cells pre-treated with the MLY cocktail. The line and arrow indicate the threshold intensity levels applied to distinguish cells that express the linker and cells that do not. Each data point represents an ROI measurement, and the squares are binned values of expression level and anisotropy, false colored for the control (green) and MLY-treated (red). **C**) Intensity (left and middle) and anisotropy (right) images of YFP-GPI cells, co-expressing mCherry-LacC2-EzAFBD (middle) exhibiting sensitivity to the MLY cocktail. Scale bars for A and C, 10 µm.

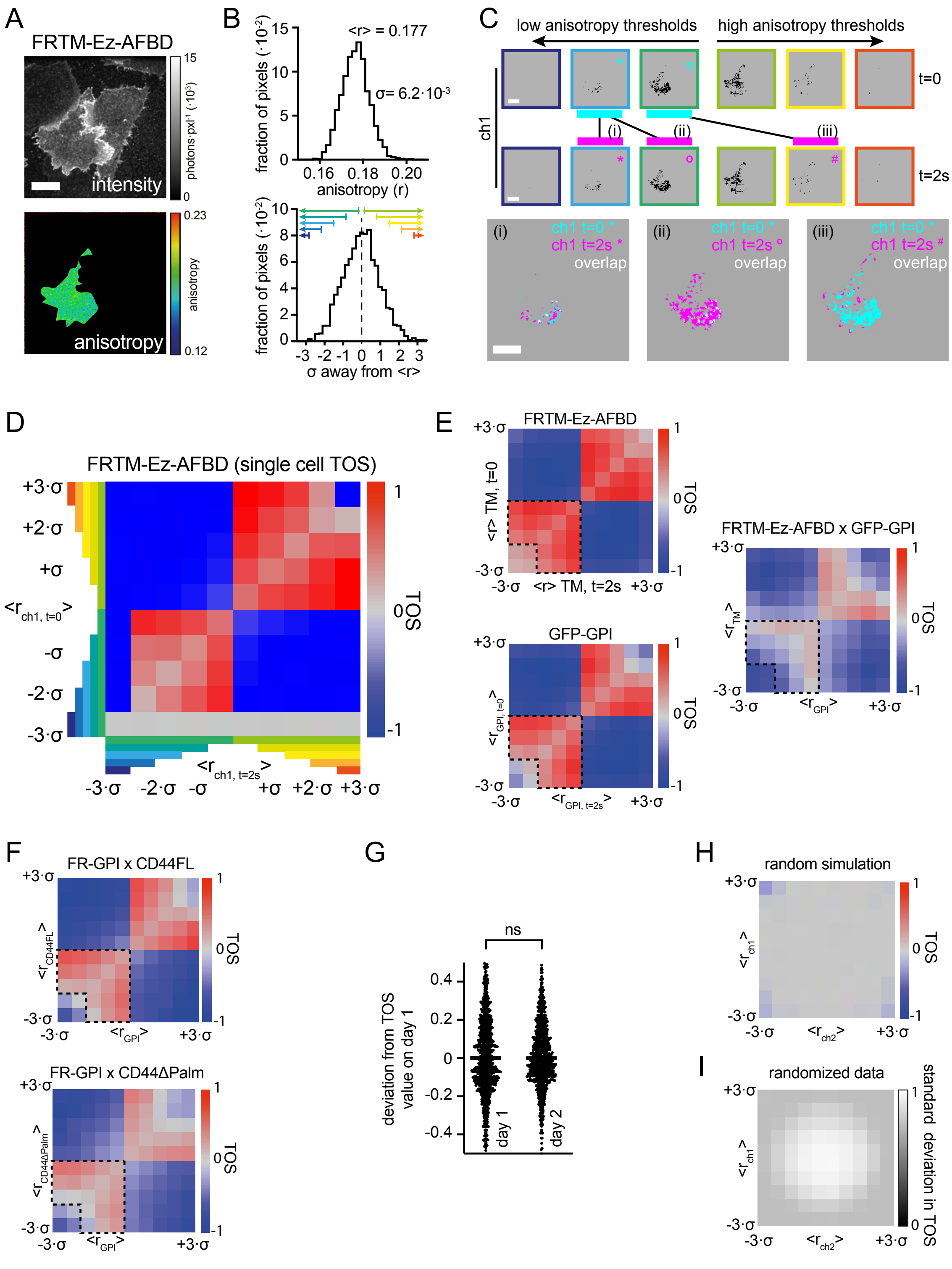


**Supplementary Figure S5. Threshold Overlap Score (TOS) analysis. A**) Total intensity image (top panel) and anisotropy image (bottom panel). The total intensity image shows the entire field of view whereas the anisotropy image displays the selection of the cell of interest that is taken forward for analysis. **B**) The distribution of anisotropy values from the selected cell from a (top panel). The distribution is shifted with respect to the mean anisotropy value and normalized to the

standard deviation of the original distribution (bottom panel). **C**) Example thresholded images showing selected pixels (black on a gray background) from the same channel at the two different timepoints (top and bottom row). The designated threshold values from low to high anisotropy regions (left to right: colored frames are correlated to colored arrows in B) result in different sets of pixels being selected. Examples of overlap between designated thresholds for the two timepoints of FRTM-Ez-AFBD in the same cell are displayed in panels i-iii. **D**) TOS-matrix of the cell selected in a resulting from the calculation of the TOS-values for all the threshold combinations. **E**) Averaged TOS matrix from multiple cells (58 cells, n=2) from temporal FRTM-Ez-AFBD correlation, temporal GPI-AP correlations and the FRTM-Ez-AFBD with GPI-AP crosscorrelation. **F**) Average TOS matrix for FR-GPI with CD44FL-GFP (52 cells, n=2) and FR-GPI with CD44ΔPalm-GFP (48 cells, n=2). The area enclosed by the dashed lines in e and f was used to calculate the mean TOS-values per cell reported in Fig 5**E** and **L**. **G**) Day to day variability of mean TOS-values normalized to day 1 (day 1: 1020 values from 255 cells, and day 2: 876 values from 219 cells) from any of the four quadrants in a TOS matrix. **H**) TOS-matrix from simulations of random spatially distributed anisotropy values. **I**) Matrix of the standard deviation in TOS-values derived from a randomized distribution of the data used for Fig. 5E,L and Supplementary Fig 5E,F. Scale bars, 10 μm for A and C. ns correspond to *p*-values of >0.05.

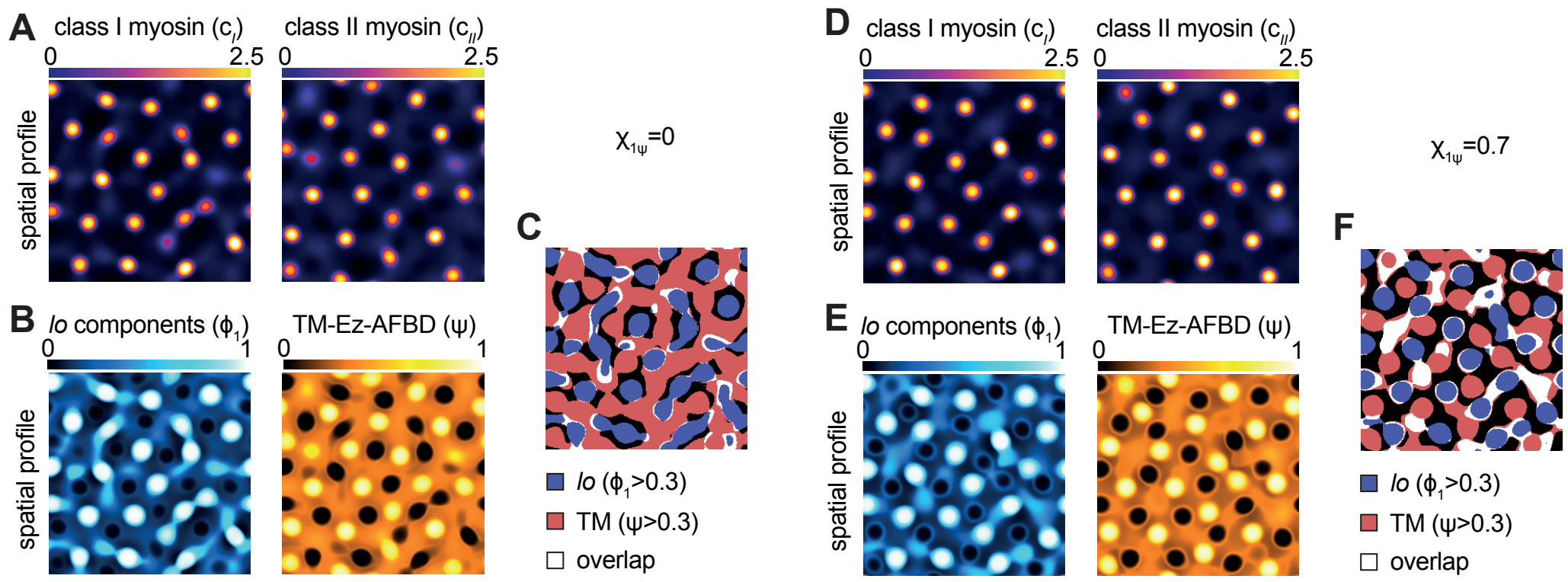


**Supplementary Figure S6. Numerical simulations for active Flory-Huggin theory. A and D)** Concentrations of the active myosin species: class I myosin ($c_1$; left) and class II myosin ($c_2$; right). **B and E)** Area fractions of the *lo* phase ($\Phi_1$; left) and the TM-Ez-AFBD ($\Psi$; right) due to their interaction with the active stresses generated by the myosin distribution displayed in a and d. **C and F)** Mesoscale domain overlap, where domains are identified based on their area fractions >0.3. Simulation corresponding to a-c used the interaction parameter $\chi_{1\psi} = 0$ and the simulations corresponding to d-f used $\chi_{1\psi} = 0.7$.

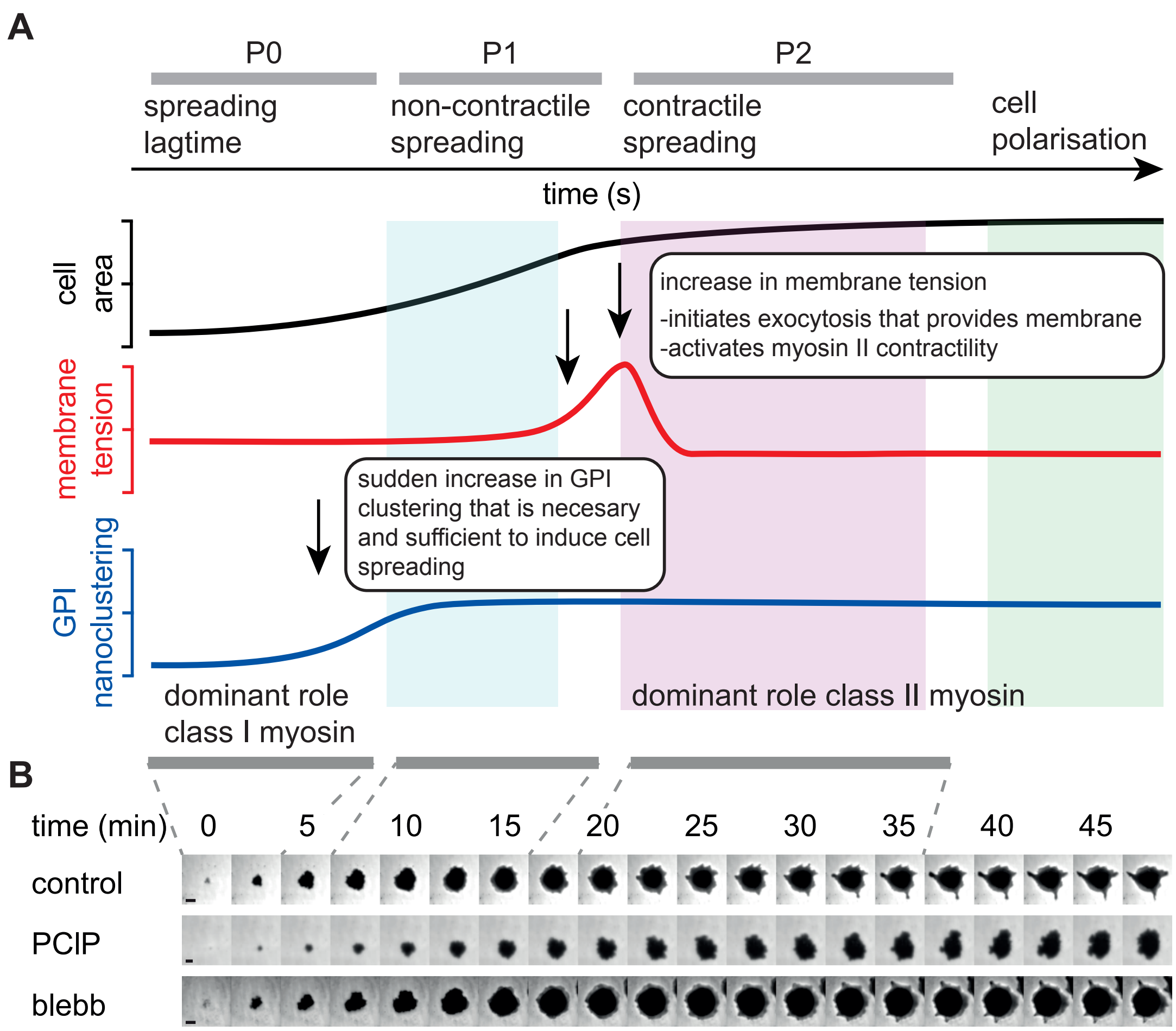


**Supplementary Figure S7. Functional consequences of myosin class independence on membrane patterning on cell spreading. A)** Schematic showing the different phases of cell spreading, their relation to GPI-AP nanoclustering and membrane tension changes following published studies and the periods of expected contributions from class I and class II myosin motor activity(*49–52*). **B)** Image montages (top) and temporal evolution of spread area (bottom) of cells treated with vehicle (DMSO, 1 hour), PCIP (5 µM, 1 hour) or Blebbistatin (blebb 50 µM, 1 hour) and adhering to fibronectin-coated surfaces imaged using Interference Reflection Microscopy.

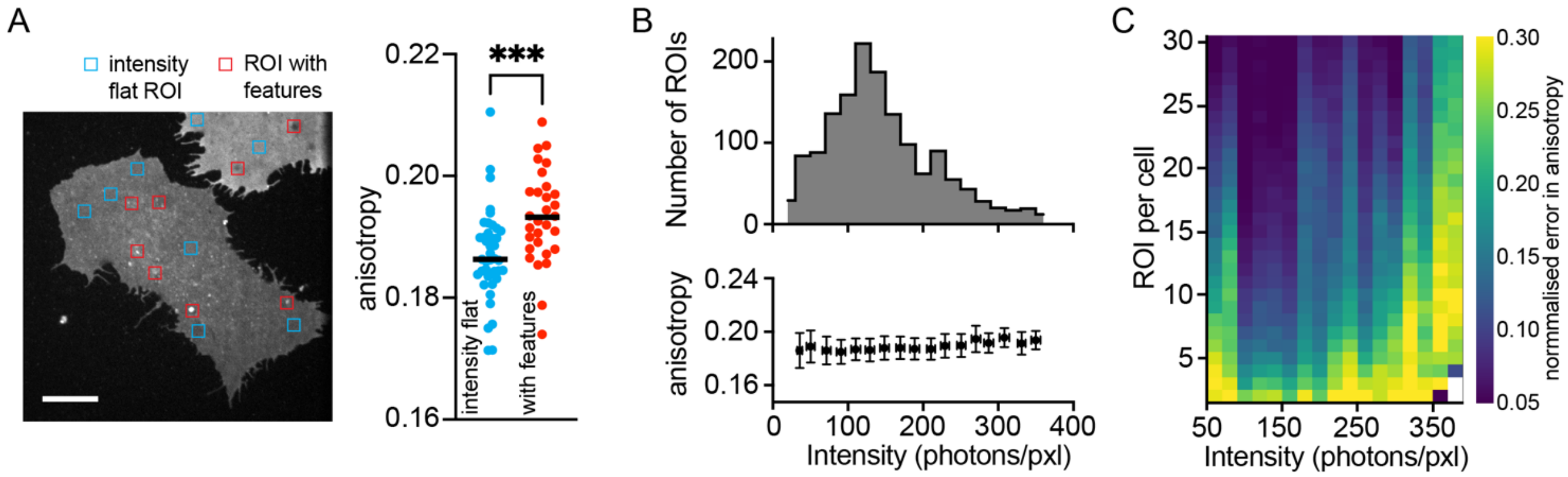


**Supplementary Figure S8: Region of interest assignment. A)** Total intensity images of two cells expressing GFP-GPI. The positions of the ROIs are chosen on areas that exhibit uniform intensity features (cyan ROIs), ie lack both high or low values compared to the mean value. The signal from these areas is expected to come from ROIs that represent a single plasma membrane sheet. Regions that contain intensity features (red ROIs) will be contaminated with signal coming from additional nearby features such as endosomes or membrane protrusions or indentations. Their distinct nature is revealed by comparing the anisotropy. **B)** A dataset from 46 cells where more than 30 ROIs have been sampled from each cell (1491 ROIs in total), shows the expected intensity independence of GFP-GPI anisotropy (bottom panel). The majority of the ROIs cover the mid-intensity range (top panel). **C)** To calculate the error associated in choosing a set number of ROIs ($N_{ROI}$) we iterated 500 random $N_{ROI}$ ROI choices from each cell in the dataset. The normalized error associated with the number of ROIs quickly falls below 0.2 for $N_{ROI} > 5$ at the mid-intensity section. In order to maintain limited error for the entire intensity range of the data we have chosen to sample on average 10 ROIs per cell.

## Supplementary Table 1. List of constructs

| Construct name | Description | Vector / primer / Restriction site / Resistance | Source |
|---|---|---|---|
| CD44-GFP | Mouse CD44 standard isoform tagged to GFP | pEGFPN1; Kan, BglII, EcoRI sites | (*30*,*69*) |
| CD44ΔPalm-GFP | Mouse CD44(C290A,C299A) defective in palmitoylation | pEGFPN1; Kan, BglII, EcoRI sites | (*30*) |
| EGFP-GPI(M3GG) | EGFP tagged to the GPI signal of the folate receptor | pJB20; Amp | (*70*) |
| FR-GPI | Human folate receptor | pMEP4; Amp | (*36*) |
| YFP-GPI | YFP tagged to GPI anchor | pEGFP-N1(GFP replaced by YFP); Kan | (*32*) |
| mRuby GPI | EGFP replaced by mRuby in M3GG | pJB20; Amp | (*51*) |
| mCherry-LactC2-EzAFBBD | mCherry-LactC2 (PS binding domain) fused with actin binding domain of Ezrin | pcDNA3.1, Amp, cloned using Gibson assembly | In this study, Main Text pg 11, lines 406-15. |
| FRTM-Ez-AFBD | Folate Receptor tagged to TM-domain of IgG and AFBD of Ezrin | pcDNA3.1; Amp; HindIII and NotI | (*14*) |
| FRTM-Ez-AFBD* | Single point mutation in AFBD domain of the FRTM-Ez-AFBD proteins to reduce actin binding | pcDNA3.1; Amp; HindIII and NotI | (*14*) |
| GFP-Myo1c | Wildtype Myosin1c isoform (mouse) | pEGFP-N1, Kan | (*29*) |
| GFP-Myo1c-R903A | Myosin1c isoform (mouse) with R→A mutation that is defective in PIP2 lipid binding (R903A) | pEGFP-N1, Kan | (*29*) |
| GFP-Myo1b | Wildtype Myosin1b isoform (mouse) | Kan | (*71*) |
| mCherry-Myo2a | Wild type non-muscle Myosin2a tagged to mCherry | Kan | Gift from Mike Sheetz |

## Supplementary Table 2. List of Cell Lines

| Cell lines | Description | Source |
|---|---|---|
| GG8 | CHO-K1 cells (TRVb-1), devoid of transferrin receptor (TfR) and stably transfected with human TfR and EGFP-GPI | (*32*) |
| FR-GPI | CHO-K1 cells (TRVb-1), devoid of transferrin receptor (TfR) and stably transfected with human TfR and FR-GPI | (*32*) |
| MYG1; m-YFP-GPI expressing CHO cells | CHO-K1 cells (TRVb-1), devoid of transferrin receptor (TfR) and stably transfected with human TfR and YFP-GPI | (*32*) |
| CHO-FRTM-Ez-AFBD | CHO-K1 cells (TRVb-1), devoid of transferrin receptor (TfR) and stably transfected with FRTM-Ez-AFBD | (*14*) |
| CHO-FRTM-Ez-AFBD* | CHO-K1 cells (TRVb-1), devoid of transferrin receptor (TfR) and stably transfected with FRTM-Ez-AFBD* | (*14*) |
| FR-AGS | Human AGS cells stably transfected with FR-GPI | (*51*) |
| U2OS-GG | U2OS cells stably transfected with GFP tagged to GPI anchor | (*51*) |

## Supplementary Table 3. List of fly-lines

| Name | Description | Source |
|---|---|---|
| w[1118] | control | Bloomington |
| UAS GPI::GFP | Gift from Susan Eaton | (*72*) |
| Gal4 collagen | Gift from C Dearolf | (*72*) |
| w[1118];P{GD1695}v7916 | sqh (CG3595) dsRNAi under UAS promotor | VRDC |
| w[1118];P{GD1566}v7819 | zip (CG15792) dsRNAi under UAS promotor | VRDC |
| P{KK102456}VIE-260B | myo31DF (CG7438) dsRNAi under UAS promotor | VRDC |
| P{KK101033}VIE-260B | myo61F (CG9155) dsRNAi under UAS promotor | VRDC |

**Supplementary Table 4. List of Reagents**

| Name | Source |
|---|---|
| Fetal Bovine Serum (FBS) | Gibco, 16000044 |
| Ham’s F12 media | HiMedia AT144-5L |
| penicillin-streptomycin-glutamine cocktail | Sigma, G1146 |
| TrypL Express | Thermo Fisher Scientific 12-604-039 |
| Fugene6 | Promega, E2692 |
| McCoy’s 5A | HiMedia AT057A |
| Human Fibronectin | Merck Millipore |
| Helmanex III | Hellma Analytics |
| PLB | Synthesized by PP Singh CSIR-IIIM, Jammu, India |
| DharmaFECT | T-2001-02 |
| ON-TARGETplus Non-targeting Pool | D-001810-10-05 (Dharmacon) |
| MYO1B SMART siRNA pool | L-023110-01-0005 (Dharmacon) |
| MYO1C SMART siRNA pool | L-015121-00-0005 (Dharmacon) |
| MYO1D SMART siRNA pool | L-023316-01-0005 (Dharmacon) |
| MemBrite Fix 405/430 | Biotium, 30092-T |
| Phalloidin Abberior Star 635 | Sigma, 30972 |
| Myosin 1B Antibody | Novus Biologicals,NBP1-87739, RRID:AB_11025957 |
| Myosin 1C Antibody | Novus Biologicals,NBP1-87745, RRID:AB_11035945 |
| Myosin 1D (E6TT6Q) antibody | Cell Signaling Technology, 90307s |
| Anti-Beta Tubulin antibody (clone, AA2) | Sigma-Aldrich, T8328, RRID:AB_1844090 |
| Pierce microBCA protein assay kit and reagents | Thermo Scientific, 23235 |
| cOmplete™, EDTA-free Protease Inhibitor Cocktail | Millipore Sigma, 11873580001 |
| Ripa Buffer (10X) | Cell Signaling Technology, 9806S |
| Immobilon-P PVDF Membrane | Merck, IPVH00010 |
| EveryBlot Blocking Buffer | Bio-Rad, 12010020 |
| Clarity Max™ Western ECL Substrate | Bio-Rad, 1705062 |
| MβCD | Sigma Aldrich, C4555 |
| Jasplakinolide | Invitrogen, J7473 |
| Blebbistatin | Sigma Aldrich, B0560 |
| Y-27632 | Sigma Aldrich, Y0503 |
| ML7 | Sigma Aldrich, I2764 |
| Pentachloropseudilin (PCIP) | Synthesized in the lab of PP Singh and Ram V. CSIR-IIIM, Jammu, India (Ref 39) |

## Supplementary Table 5: Qualitative effect of simulation parameters

| Changes in AFH parameters | Physical interpretation of parameters | Effect on segregation of *lo* component and TM-EzAFBD |
|---|---|---|
| Increase in $B_{I,II}$ | Competitive binding between class I and class II myosin | Decreases domain overlap |
| Increase in $Q$ | Competitive inhibition of active stresses | Decreases domain overlap |
| Increase in $\chi_{2\psi}$ | Affinity between *ld* component and TM-EzAFBD | Increases domain overlap |
| Increase in $\chi_{1\psi}$ | Affinity between *lo* component and TM-EzAFBD | Promotes segregation and decreases domain overlap |
| Increase in $K_I$, $K_{II}$ | Characterizes coupling between class I myosin with *lo* component and class II myosin with TM-EzAFBD | Promotes nano- and mesoscale segregation of *lo* components and TM-EzAFBD |

## REFERENCES


1. J. C. M. Holthuis, A. K. Menon, Lipid landscapes and pipelines in membrane homeostasis. *Nature* **510**, 48–57 (2014).

2. T. Kobayashi, A. K. Menon, Transbilayer lipid asymmetry. *Curr. Biol.* **28**, R386–R391 (2018).

3. S. Mayor, A. Bhat, A. Kusumi, A survey of models of cell membranes: Toward a new understanding of membrane organization. *Cold Spring Harb. Perspect. Biol.* **15**, a041394 (2023).

4. S. A. Shelby, S. L. Veatch, The membrane phase transition gives rise to responsive plasma membrane structure and function. *Cold Spring Harb. Perspect. Biol.* **15**, a041394 (2023).

5. J. M. Kalappurakkal, P. Sil, S. Mayor, Toward a new picture of the living plasma membrane. *Protein Sci.* **29**, 1355–1365 (2020).

6. M. F. Garcia-Parajo, A. Cambi, J. A. Torreno-Pina, N. Thompson, K. Jacobson, Nanoclustering as a dominant feature of plasma membrane organization. *J. Cell Sci.* **127**, 4995–5005 (2014).

7. K. Jacobson, P. Liu, B. C. Lagerholm, The lateral organization and mobility of plasma membrane components. *Cell* **177**, 806–819 (2019).

8. D. Lingwood, K. Simons, Lipid rafts as a membrane-organizing principle. *Science* **327**, 46–50 (2010).

9. K. Simons, E. Ikonen, Functional rafts in cell membranes. *Nature* **387**, 569–572 (1997).

10. S. L. Veatch, N. Rogers, A. Decker, S. A. Shelby, The plasma membrane as an adaptable fluid mosaic. *Biochim. Biophys. Acta Biomembr.* **1865**, 184114 (2023).

11. S. A. Shelby, I. Castello-Serrano, K. C. Wisser, I. Levental, S. L. Veatch, Membrane phase separation drives responsive assembly of receptor signaling domains. *Nat. Chem. Biol.* **19**, 750–758 (2023).

12. E. Sezgin, I. Levental, S. Mayor, C. Eggeling, The mystery of membrane organization: Composition, regulation and roles of lipid rafts. *Nat. Rev. Mol. Cell Biol.* **18**, 361–374 (2017).

13. K. Gaus, E. Chklovskaia, B. Fazekas de St, W. Groth, T. H. Jessup, T. Harder, Condensation of the plasma membrane at the site of T lymphocyte activation. *J. Cell Biol.* **171**, 121–131 (2005).

14. K. Gowrishankar, S. Ghosh, S. Saha, R. C, S. Mayor, M. Rao, Active remodeling of cortical actin regulates spatiotemporal organization of cell surface molecules. *Cell* **149**, 1353–1367 (2012).

15. D. Goswami, K. Gowrishankar, S. Bilgrami, S. Ghosh, R. Raghupathy, R. Chadda, R. Vishwakarma, M. Rao, S. Mayor, Nanoclusters of GPI-anchored proteins are formed by cortical actin-driven activity. *Cell* **135**, 1085–1097 (2008).

16. S. Saha, A. Das, C. Patra, A. A. Anilkumar, P. Sil, S. Mayor, M. Rao, Active emulsions in living cell membranes driven by contractile stresses and transbilayer coupling. *Proc. Natl. Acad. Sci. U.S.A.* **119**, e2123056119 (2022).

17. D. V. Köster, K. Husain, E. Iljazi, A. Bhat, P. Bieling, R. D. Mullins, M. Rao, S. Mayor, Actomyosin dynamics drive local membrane component organization in an in vitro active composite layer. *Proc. Natl. Acad. Sci. U.S.A.* **113**, E1645–E1654 (2016).

18. S. J. Plowman, C. Muncke, R. G. Parton, J. F. Hancock, H-ras, K-ras, and inner plasma membrane raft proteins operate in nanoclusters with differential dependence on the actin cytoskeleton. *Proc. Natl. Acad. Sci. U.S.A.* **102**, 15500–15505 (2005).

19. R. Raghupathy, A. A. Anilkumar, A. Polley, P. P. Singh, M. Yadav, C. Johnson, S. Suryawanshi, V. Saikam, S. D. Sawant, A. Panda, Z. Guo, R. A. Vishwakarma, M. Rao, S. Mayor, Transbilayer lipid interactions mediate nanoclustering of lipid-anchored proteins. *Cell* **161**, 581–594 (2015).

20. M. Rao, S. Mayor, Active organization of membrane constituents in living cells. *Curr. Opin. Cell Biol.* **29**, 126–132 (2014).

21. A. Sebé-Pedrós, X. Grau-Bové, T. A. Richards, I. Ruiz-Trillo, Evolution and classification of myosins, a paneukaryotic whole-genome approach. *Genome Biol. Evol.* **6**, 290–305 (2014).

22. M. A. Hartman, D. Finan, S. Sivaramakrishnan, J. A. Spudich, Principles of unconventional myosin function and targeting. *Annu. Rev. Cell Dev. Biol.* **27**, 133–155 (2011).

23. G. Lebreton, C. Géminard, F. Lapraz, S. Pyrpassopoulos, D. Cerezo, P. Spéder, E. M. Ostap, S. Noselli, Molecular to organismal chirality is induced by the conserved myosin 1D. *Science* **362**, 949–952 (2018).

24. S. Tojkander, G. Gateva, P. Lappalainen, Actin stress fibers—Assembly, dynamics and biological roles. *J. Cell Sci.* **125**, 1855–1864 (2012).

25. S. Banerjee, M. L. Gardel, U. S. Schwarz, The actin cytoskeleton as an active adaptive material. *Annu. Rev. Condens. Matter Phys.* **11**, 421–439 (2020).

26. A. Bansal, A. Das, M. Rao, Active segregation dynamics in the living cell. *Indian J. Phys.* **96**, 2567–2576 (2022).

27. B. B. McIntosh, E. M. Ostap, Myosin-I molecular motors at a glance. *J. Cell Sci.* **129**, 2689 (2016).

28. B. A. Truong Quang, R. Peters, D. A. D. Cassani, P. Chugh, A. G. Clark, M. Agnew, G. Charras, E. K. Paluch, Extent of myosin penetration within the actin cortex regulates cell surface mechanics. *Nat. Commun.* **12**, 6511 (2021).

29. D. E. Hokanson, J. M. Laakso, T. Lin, D. Sept, E. M. Ostap, Myo1c binds phosphoinositides through a putative pleckstrin homology domain. *Mol. Biol. Cell* **17**, 4856–4865 (2006).

30. P. Sil, N. Mateos, S. Nath, S. Buschow, C. Manzo, K. G. N. Suzuki, T. Fujiwara, A. Kusumi, M. F. Garcia-Parajo, S. Mayor, Dynamic actin-mediated nano-scale clustering of CD44

regulates its meso-scale organization at the plasma membrane. *Mol. Biol. Cell* **31**, 561–579 (2020).

31. R. Chandran, G. Kale, J. M. Philippe, T. Lecuit, S. Mayor, Distinct actin-dependent nanoscale assemblies underlie the dynamic and hierarchical organization of E-cadherin. *Curr. Biol.* **31**, 1726–1736.e4 (2021).

32. P. Sharma, R. Varma, R. C. C. Sarasij, K. Gousset, G. Krishnamoorthy, M. Rao, S. Mayor, K. Ira, G. Gousset, M. Krishnamoorthy, S. M. Rao, K. Ira, G. Gousset, M. Krishnamoorthy, S. M. Rao, Nanoscale organization of multiple GPI-anchored proteins in living cell membranes. *Cell* **116**, 577–589 (2004).

33. M. Kovács, J. Tóth, C. Hetényi, A. Málnási-Csizmadia, J. R. Sellers, Mechanism of blebbistatin inhibition of myosin II. *J. Biol. Chem.* **279**, 35557–35563 (2004).

34. T. S. van Zanten, P. S. Greeshma, S. Mayor, Quantitative fluorescence emission anisotropy microscopy for implementing homo–fluorescence resonance energy transfer measurements in living cells. *Mol. Biol. Cell* **34**, tp1 (2023).

35. G. Totsukawa, Y. Wu, Y. Sasaki, D. J. Hartshorne, Y. Yamakita, S. Yamashiro, F. Matsumura, Distinct roles of MLCK and ROCK in the regulation of membrane protrusions and focal adhesion dynamics during cell migration of fibroblasts. *J. Cell Biol.* **164**, 427–439 (2004).

36. R. Varma, S. Mayor, GPI-anchored proteins are organized in submicron domains at the cell surface. *Nature* **394**, 798–801 (1998).

37. M. Kumar, S. Sharma, P. Sil, M. Kushwaha, S. Mayor, R. A. Vishwakarma, P. P. Singh, C–H arylation of N-heteroarenes under metal-free conditions and its application towards the synthesis of pentabromo- and pentachloropseudilins. *Eur. J. Org. Chem.* **2019**, 3591–3598 (2019).

38. K. Chinthalapudi, M. H. Taft, R. Martin, S. M. Heissler, M. Preller, F. K. Hartmann, H. Brandstaetter, J. Kendrick-Jones, G. Tsiavaliaris, H. O. Gutzeit, R. Fedorov, F. Buss, H. J.

Knölker, L. M. Coluccio, D. J. Manstein, Mechanism and specificity of pentachloropseudilin-mediated inhibition of myosin motor activity. *J. Biol. Chem.* **286**, 29700–29708 (2011).

39. J. D. Diaz-Valencia, L. A. Estrada-Abreo, L. Rodríguez-Cruz, A. R. Salgado-Aguayo, G. Patiño-López, Class I myosins, molecular motors involved in cell migration and cancer. *Cell Adh. Migr.* **16**, 1 (2022).

40. M. J. Tyska, A. T. Mackey, J. D. Huang, N. G. Copeland, N. A. Jenkins, M. S. Mooseker, Myosin-1a is critical for normal brush border structure and composition. *Mol. Biol. Cell* **16**, 2443–2457 (2005).

41. D. E. Hokanson, E. M. Ostap, Myo1c binds tightly and specifically to phosphatidylinositol 4,5-bisphosphate and inositol 1,4,5-trisphosphate. *Proc. Natl. Acad. Sci. U.S.A.* **103**, 3118–3123 (2006).

42. J. Abramson, J. Adler, J. Dunger, R. Evans, T. Green, A. Pritzel, O. Ronneberger, L. Willmore, A. J. Ballard, J. Bambrick, S. W. Bodenstein, D. A. Evans, C. C. Hung, M. O'Neill, D. Reiman, K. Tunyasuvunakool, Z. Wu, A. Žemgulytė, E. Arvaniti, C. Beattie, O. Bertolli, A. Bridgland, A. Cherepanov, M. Congreve, A. I. Cowen-Rivers, A. Cowie, M. Figurnov, F. B. Fuchs, H. Gladman, R. Jain, Y. A. Khan, C. M. R. Low, K. Perlin, A. Potapenko, P. Savy, S. Singh, A. Stecula, A. Thillaisundaram, C. Tong, S. Yakneen, E. D. Zhong, M. Zielinski, A. Žídek, V. Bapst, P. Kohli, M. Jaderberg, D. Hassabis, J. M. Jumper, Accurate structure prediction of biomolecular interactions with AlphaFold 3. *Nature* **630**, 493–500 (2024).

43. K. E. Blow, M. Parrag, P. J. Stansfeld, CCD2MD: A suite of packages for preparing co-folded outputs for molecular dynamics simulations. *J. Chem. Inf. Model.* **65**, 12145–12154 (2025).

44. H. Sheng, W. Stauffer, H. N. Lim, Systematic and general method for quantifying localization in microscopy images. *Biol. Open* **5**, 1882–1893 (2016).

45. T. S. van Zanten, A. Cambi, M. Koopman, B. Joosten, C. G. Figdor, M. F. Garcia-Parajo, Hotspots of GPI-anchored proteins and integrin nanoclusters function as nucleation sites for cell adhesion. *Proc. Natl. Acad. Sci. U.S.A.* **106**, 18557–18562 (2009).

46. Z. Ma, S. Shi, M. Ren, C. Pang, Y. Zhan, H. An, F. Sun, Molecular mechanism of CD44 homodimerization modulated by palmitoylation and membrane environments. *Biophys. J.* **121**, 2671–2683 (2022).

47. F. Sun, C. F. E. Schroer, C. R. Palacios, L. Xu, S.-Z. Luo, S. J. Marrink, Molecular mechanism for bidirectional regulation of CD44 for lipid raft affiliation by palmitoylations and PIP2. *PLoS Comput. Biol.* **16**, e1007777 (2020).

48. I. S. Babina, E. A. McSherry, S. Donatello, A. D. Hill, A. M. Hopkins, A novel mechanism of regulating breast cancer cell migration via palmitoylation-dependent alterations in the lipid raft affiliation of CD44. *Breast Cancer Res.* **16**, R19 (2014).

49. H. Oh, H. Kim, B. Shin, K. H. Lee, M. G. Yeo, W. K. Song, Interaction of Crk with myosin-1c participates in fibronectin-induced cell spreading. *Int. J. Biol. Sci.* **9**, 778–791 (2013).

50. B. Pontes, P. Monzo, L. Gole, A.-L. Le Roux, A. J. Kosmalska, Z. Y. Tam, W. Luo, S. Kan, V. Viasnoff, P. Roca-Cusachs, L. Tucker-Kellogg, N. C. Gauthier, Membrane tension controls adhesion positioning at the leading edge of cells. *J. Cell Biol.* **216**, 2959–2977 (2017).

51. J. M. Kalappurakkal, A. A. Anilkumar, C. Patra, T. S. van Zanten, M. P. Sheetz, S. Mayor, Integrin mechano-chemical signaling generates plasma membrane nanodomains that promote cell spreading. *Cell* **177**, 1738–1756.e23 (2019).

52. Y. Cai, N. Biais, G. Giannone, M. Tanase, G. Jiang, J. M. Hofman, C. H. Wiggins, P. Silberzan, A. Buguin, B. Ladoux, M. P. Sheetz, Nonmuscle myosin IIA-dependent force inhibits cell spreading and drives F-actin flow. *Biophys. J.* **91**, 3907–3920 (2006).

53. B. N. Manz, B. L. Jackson, R. S. Petit, M. L. Dustin, J. T. Groves, Dissecting T cell receptor nanocluster signaling. *Biophys. J.* **96**, 3596 (2009).

54. M. J. Taylor, K. Husain, Z. J. Gartner, S. Mayor, R. D. Vale, A DNA-based T cell receptor reveals a role for receptor clustering in ligand discrimination. *Cell* **169**, 108–119.e20 (2017).

55. B. A. Truong Quang, M. Mani, O. Markova, T. Lecuit, P. F. Lenne, Principles of E-cadherin supramolecular organization in vivo. *Curr. Biol.* **23**, 2197–2207 (2013).

56. P. Kanchanawong, D. A. Calderwood, Organization, dynamics and mechanoregulation of integrin-mediated cell–ECM adhesions. *Nat. Rev. Mol. Cell Biol.* **24**, 142–161 (2022).

57. M. F. Garcia-Parajo, S. Mayor, The ubiquitous nanocluster: A molecular scale organizing principle that governs cellular information flow. *Curr. Opin. Cell Biol.* **86**, 102285 (2024).

58. M.-T. Prospéri, P. Lépine, F. Dingli, P. Paul-Gilloteaux, R. Martin, D. Loew, H.-J. Knölker, E. Coudrier, Myosin 1b functions as an effector of EphB signaling to control cell repulsion. *J. Cell Biol.* **210**, 347–361 (2015).

59. J. Gerhart, M. Kirschner, The theory of facilitated variation. *Proc. Natl. Acad. Sci. U.S.A.* **104**, 8582–8589 (2007).

60. R. Heintzmann, P. K. Relich, R. P. J. Nieuwenhuizen, K. A. Lidke, B. Rieger, Calibrating photon counts from a single image. arXiv:1611.05654 [physics.ins-det] (2016).

61. F. Huang, T. M. P. Hartwich, F. E. Rivera-Molina, Y. Lin, W. C. Duim, J. J. Long, P. D. Uchil, J. R. Myers, M. A. Baird, W. Mothes, M. W. Davidson, D. Toomre, J. Bewersdorf, Video-rate nanoscopy using sCMOS camera–specific single-molecule localization algorithms. *Nat. Methods* **10**, 653–658 (2013).

62. S. S. Vogel, T. A. Nguyen, B. W. van der Meer, P. S. Blank, The impact of heterogeneity and dark acceptor states on FRET: implications for using fluorescent protein donors and acceptors. *PLOS ONE* **7**, e49593 (2012).

63. D. Tsygankov, P.-H. Chu, H. Chen, T. C. Elston, K. M. Hahn, User-friendly tools for quantifying the dynamics of cellular morphology and intracellular protein clusters. *Methods Cell Biol.* **123**, 409–427 (2014).

64. A. K. Bansal, M. Rao, Nonequilibrium asymmetry in the living cell membrane. *Faraday Discuss.* **259**, 60–82 (2025).

65. H. Tanaka, Viscoelastic phase separation in soft matter and foods. *Faraday Discuss.* **158**, 371 (2012).

66. M. Doi, A. Onuki, Dynamic coupling between stress and composition in polymer solutions and blends. *J. Phys. II* **2**, 1631–1656 (1992).

67. M. C. Marchetti, J. F. Joanny, S. Ramaswamy, T. B. Liverpool, J. Prost, M. Rao, R. A. Simha, Hydrodynamics of soft active matter. *Rev. Mod. Phys.* **85**, 1143–1189 (2013).

68. F. Sievers, A. Wilm, D. Dineen, T. J. Gibson, K. Karplus, W. Li, R. Lopez, H. McWilliam, M. Remmert, J. Söding, J. D. Thompson, D. G. Higgins, Fast, scalable generation of high-quality protein multiple sequence alignments using Clustal Omega. *Mol. Syst. Biol.* **7**, 539 (2011).

69. P. Mrass, I. Kinjyo, L. G. Ng, S. L. Reiner, E. Puré, W. Weninger, CD44 mediates successful interstitial navigation by killer T cells and enables efficient antitumor immunity. *Immunity* **29**, 971–985 (2008).

70. S. Paladino, D. Sarnataro, R. Pillich, S. Tivodar, L. Nitsch, C. Zurzolo, Protein oligomerization modulates raft partitioning and apical sorting of GPI-anchored proteins. *J. Cell Biol.* **167**, 699–709 (2004).

71. C. G. Almeida, A. Yamada, D. Tenza, D. Louvard, G. Raposo, E. Coudrier, Myosin 1b promotes the formation of post-Golgi carriers by regulating actin assembly and membrane remodelling at the trans-Golgi network. *Nat. Cell Biol.* **13**, 779–789 (2011).

72. V. Greco, M. Hannus, S. Eaton, Argosomes: A potential vehicle for the spread of morphogens through epithelia. *Cell* **106**, 633–645 (2001).
